# Bioindicator “fingerprints” of methane-emitting thermokarst features in Alaskan soils

**DOI:** 10.3389/fmicb.2024.1462941

**Published:** 2025-02-21

**Authors:** Chuck R. Smallwood, Nicholas Hasson, Jihoon Yang, Jenna Schambach, Haley Bennett, Bryce Ricken, Jason Sammon, Monica Mascarenas, Naomi Eberling, Stephanie Kolker, Joshua Whiting, Wittney D. Mays, Katey Walter Anthony, Philip R. Miller

**Affiliations:** ^1^Department of Environmental Systems Biology, Sandia National Laboratories, Albuquerque, NM, United States; ^2^Water and Environmental Research Center, University of Alaska Fairbanks, Fairbanks, AK, United States; ^3^Bioresource & Environmental Security, Sandia National Laboratories, Livermore, CA, United States; ^4^Biological & Chemical Sensors, Sandia National Laboratories, Albuquerque, NM, United States; ^5^Systems Biology, Sandia National Laboratories, Livermore, CA, United States

**Keywords:** volatile organic compounds, permafrost thaw, methanogens, methanotrophs, methane emissions, anaerobic degradation, carbon sequestration, greenhouse gases

## Abstract

Permafrost thaw increases the bioavailability of ancient organic matter, facilitating microbial metabolism of volatile organic compounds (VOCs), carbon dioxide, and methane (CH_4_). The formation of thermokarst (thaw) lakes in icy, organic-rich Yedoma permafrost leads to high CH_4_ emissions, and subsurface microbes that have the potential to be biogeochemical drivers of organic carbon turnover in these systems. However, to better characterize and quantify rates of permafrost changes, methods that further clarify the relationship between subsurface biogeochemical processes and microbial dynamics are needed. In this study, we investigated four sites (two well-drained thermokarst mounds, a drained thermokarst lake, and the terrestrial margin of a recently formed thermokarst lake) to determine whether biogenic VOCs (1) can be effectively collected during winter, and (2) whether winter sampling provides more biologically significant VOCs correlated with subsurface microbial metabolic potential. During the cold season (March 2023), we drilled boreholes at the four sites and collected cores to simultaneously characterize microbial populations and captured VOCs. VOC analysis of these sites revealed “fingerprints” that were distinct and unique to each site. Total VOCs from the boreholes included > 400 unique VOC features, including > 40 potentially biogenic VOCs related to microbial metabolism. Subsurface microbial community composition was distinct across sites; for example, methanogenic archaea were far more abundant at the thermokarst site characterized by high annual CH_4_ emissions. The results obtained from this method strongly suggest that ∼10% of VOCs are potentially biogenic, and that biogenic VOCs can be mapped to subsurface microbial metabolisms. By better revealing the relationship between subsurface biogeochemical processes and microbial dynamics, this work advances our ability to monitor and predict subsurface carbon turnover in Arctic soils.

## Background

Permafrost soils, like those in interior Alaska, contain the largest terrestrial pool of temperature soil organic carbon stocks on the planet; they cover ∼15% of the global land area but account for ∼60% of global soil carbon storage (Schuur et al., 2015). Unfortunately, increasing duration of permafrost thaw seasons (Jorgenson et al., 2010; Smith et al., 2010), perpetuates a positive feedback loop in which gradual or abrupt thaw events cause the active (seasonally frozen) soil layer to deepen and the size of talik sites (unfrozen ground in the permafrost area) to expand (Liljedahl et al., 2016; Kokelj et al., 2017; Farquharson et al., 2022), triggering increased carbon flux, and hydrological flow (Farquharson et al., 2022). The most widespread form of abrupt thaw is the thermokarst (land surface collapse caused by the loss of ice-rich permafrost), which commonly results in the creation of ponds or lakes. In recent decades, the number of thermokarst lakes in interior Alaska has grown by ∼40% (Walter Anthony et al., 2021), which is particularly concerning because these newly formed thermokarst lakes are derived mainly from the thaw of Yedoma (methane-rich, Pleistocene-aged permafrost), resulting in the highest recorded CH_4_ emissions from Arctic lakes (Walter Anthony et al., 2016).

Recently, high-C flux has been observed in the Arctic, but while some ecosystem models suggest that terrestrial permafrost is currently a net CO_2_ sink (range 0–0.8 Pg C-CO_2_e yr^–1^) (Walter Anthony et al., 2018), results from Arctic soil incubation studies in the laboratory suggest that permafrost soils have intrinsically short C turnover times and may switch to become a net C source in the near future, with a potential loss of 0.22–0.53 petagrams of C annually through the end of this century (Ren et al., 2024). To date, permafrost C feedback (PCF) modeling has focused on gradual, near-surface processes of C transformation in degraded permafrost (Schaefer et al., 2014; Walter Anthony et al., 2018), and these models predict that large proportions of permafrost C in Alaska are vulnerable to abrupt thaw events, on timescales of months to years (Olefeldt et al., 2016). However, our inability to consistently monitor subsurface biogeochemical cycles in the field has obscured the fine-scale biogeochemical processes that underlie thaw events (gradual or abrupt), which occur on timescales of hours to days (Turetsky et al., 2019). This gap in the experimental data significantly limits the accuracy of current PCF system models, resulting in high magnitudes of uncertainty associated with permafrost thaw (Bradford et al., 2016) and hindering our ability to predict increases in thaw depth caused by abrupt thaw events that seasonal freeze cannot overcome (Turetsky et al., 2020). To enable a detailed characterization of subsurface biogeochemical processes and constrain the uncertainty in PCF models, we urgently need more experimental data (Tao et al., 2024).

To effectively account for permafrost ecosystems, our analysis this data must effectively differentiate abiotic and biogeochemical processes, particularly because the microbes that drive biogeochemical processes often operate on shorter timescales than abiotic processes (Zolkos and Tank, 2020; Waldrop et al., 2023). Laboratory and small-scale field experiments have demonstrated that microbes respond rapidly to thaw (Mackelprang et al., 2011) and that distinct microbial species are present in frozen versus thawing permafrost (Mackelprang et al., 2011; Monteux et al., 2018; Ji et al., 2020). Although soil cores are often required to characterize microbial activity in the subsurface, the direct extraction of permafrost soil samples is destructive to fragile ecosystems. The release of unique microbial metabolites has been detected during thaw into lakes and streams, which could provide non-destructive measurements of microbial processes (Vonk et al., 2015). Moreover, volatile organic compounds (VOCs) such as methane (CH_4_) can be emitted from thawing soils (PeÑUelas et al., 2014; Schulz-Bohm et al., 2017).

Biogenic VOCs are generated by microbes (e.g., bacteria, archaea, and fungi) and range from small- (< C15) to medium- (C16–C28) chain molecules with low molecular masses (< 300 Da), high vapor pressure, and a low boiling point (Schulz-Bohm et al., 2017; Franchina et al., 2019). These molecules are typically in the gas phase but may also occur dissolved in liquid phase until environmental conditions favor evaporation. VOCs are normally lipophilic compounds capable of diffusing through water and gas-filled pores in soil environments (Effmert et al., 2012; Schmidt et al., 2016; Schulz-Bohm et al., 2017). Unlike dissolved metabolites, which are often involved in short distance (< 12 cm) infochemical biological interactions, VOCs can travel and act on other biosystems even at long distances (> 2 m) (Tyc et al., 2017; Westhoff et al., 2017; Schulz-Bohm et al., 2018). An estimated 2,000 VOCs attributed to about 1,000 microbial hosts have already been identified, and more identifications are occurring daily (Lemfack et al., 2018). VOCs can be measured from soils over time to elucidate subsurface biogeochemical processes for incorporation into process-based models to predict early warning signs of thaw (Kramshøj et al., 2019; Ghirardo et al., 2020; Li B. et al., 2021).

In this study, we sought to determine whether biogenic VOCs (1) can be effectively captured in arctic environments during winter, and (2) if this approach provides more biologically relevant chemical signatures that correlate to subsurface microbial metabolic potential during winter in perennially thawed taliks. Guided by electrical resistivity (ER) maps of subsurface taliks, we sought to validate these measurements and characterize microbiomes and VOCs related to these CH_4_-emitting taliks. Focused on four upland thermokarst sites characterized by wintertime CH_4_-emitting taliks in interior Alaska, we quantified microbial communities using short-read sequencing metagenomics and measured corresponding borehole VOCs as a means of detecting subsurface biochemical processes.

Because the boundaries of thermokarst lakes are areas where positive feedback between permafrost thaw and atmospheric warming can be actively measured (Bradford et al., 2016; in ’t Zandt et al., 2020), we took four core samples from three Yedoma-taliks north of Fairbanks, Alaska. Permafrost soil microbiomes have been shown to be dramatically different during winter, with high methanogen abundance as well as metabolic processes related to degradation of soil organic matter (Mackelprang et al., 2011; Vigneron et al., 2019), so we collected our samples in March 2023. Typically, metagenomes are used to identify microbes and genes related to carbon degradation pathways (Fierer et al., 2012). However, recent advancements in VOC collection and analysis have enabled characterization of volatile metabolite emissions from microbiomes (volatilome) (Meredith and Tfaily, 2022; Jiao et al., 2023). Thus, volatilomics can provide a means to investigate the chemical conversation between subsurface microbes in real-time and resolve the metabolic frontier of unresolved pathways. We surmised that corresponding VOCs from these distinct talik sites could provide realistic bioindicators of subsurface biogeochemical processes related to permafrost degradation.

## Materials and methods

### Geophysical surveys of drilling locations, methane flux measurements, and soil cores collection

**FIGURE 1:**
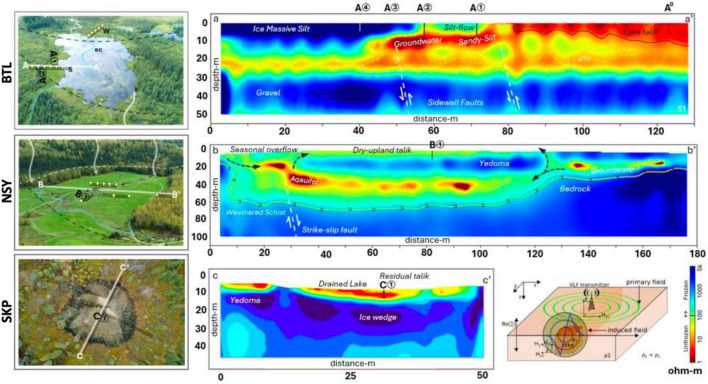
Geophysical resistivity surveys were conducted using very long frequency-magnetotelluric (VLF-MT) to generate 2D-inversion models showing cross-sections of thermokarst (TK) at Big Trail Lake (BTL) in a lowland valley, NorthStar Yedoma (NSY) on a well-drained hillslope, and Skidmore Pond SKP, now a shallow thermokarst pond. An overhead view of each site was paired with 2D-inversion models show the presence or absence of permafrost, and the color-contour plots indicates cold frozen permafrost (blue) and unfrozen features (yellow to red) conditions. The 2-D electrical resistivity (ER) profiles, supported by borehole data across transects (A-A’, B-B’, C-C’), indicate permafrost presence with < 100 ohm-m reflecting thawed conditions or talik. The darkest red (< 1 ohm-m) marks groundwater within talik or fault fractures. Color contours show permafrost (5,000–600 ohm-m) and thawed or water-saturated sediments (100–1 ohm-m). The BTL model shows south (A) and north (A’) transecting line indicating flux site measurements (A1, A2, A3, and A4) as well as the nearby Eddy-covariance tower (A0). The NSY model shows the east (B) and west (B’) transect indicating core site B1 sampled at NSY The SKP model shows the north (C) and south (C’) transect indicating core site C1 sampled at SKP.

For our preliminary geophysical surveys, we employed a multi-frequency EM system (GSM-19WV, Gem Systems Inc., Ontario, Canada, 2019) based on EM induction amplitudes corresponding to electrical resistivity (Ohm-m) at angular frequencies (ω) using the controlled-source very-long-frequency-magnetotelluric (VLF-MT) bands (McNeill and Labson, 1991; Elder et al., 2021). Leveraging ER data acquired from the VLF signals emitted from US Navy transmitters (16.8–24.8 kHz), we conducted magnetotelluric analysis (Sasaki, 1989); to enhance the precision of the geophysical mapping, we employed the VLF2dmf.v2 tool (Monteiro Santos et al., 2006) and Occam’s technique for 2D modeling (Constable et al., 1987). The resulting 2D-inversion models show cross-sections of permafrost for the BTL, NSY, and SKP sites (Figure 1). Following established methods (Elder et al., 2021), we measured linear methane fluxes with a portable 20 L chamber placed over each borehole. Air was briefly (120–150 s) recirculated through the chamber and a Los Gatos Research Micro-Portable Greenhouse Gas Analyzer (MGGA) (ABB Inc., Quebec City, CA) was used to measure CH_4_ concentration, with a measurement frequency of 10 Hz. Diffusive fluxes were calculated from the ideal gas law using chamber volume, temperature, atmospheric pressure; a least square fit regression was applied with a minimum R^2 value of 0.95 (Elder et al., 2021). At BTL, methane fluxes were collected at four sites transecting the lake (A1, A2, A3, and A4) and at the nearby Eddy-covariance tower (A0) (Figure 1; Supplementary Figure S1).

**FIGURE 2:**
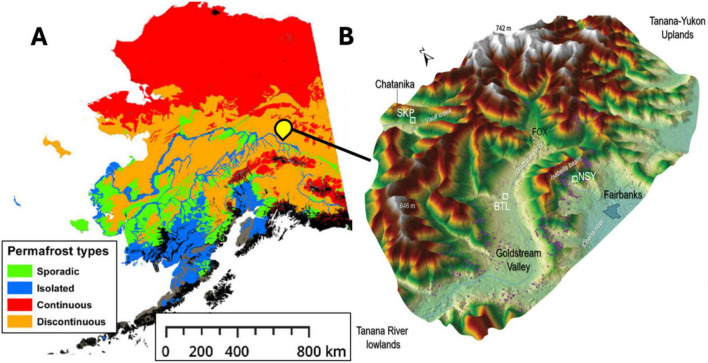
Estimates of permafrost gradients across central Alaska were adapted from Mishra and Riley (2012) showing sporadic (green), isolated (blue), continuous (red), and discontinuous (orange) permafrost generalized across Alaska **(A)**. Central Alaska Goldstream Valley area where the thermokarst sites Big Trail Lake (BTL), NorthStar Yedoma (NSY), and Skidmore Pond (SKP) sites featured in this study represent three unique watersheds near Fairbanks Alaska **(B)**.

We began by collecting four soil cores from three thermokarst systems (two well-drained thermokarst-mounds, a drained thermokarst lake, and the terrestrial margin of a recently formed thermokarst lake) (Figure 2) around Big Trail Lake (BTL), a thermokarst lake located in Goldstream valley that was formed between 1949 and 1967 (in ’t Zandt et al., 2020; Pellerin et al., 2022). The samples were taken from four Yedoma-talik sites: two on the south side of BTL boundary (BTL1, closer to the terrestrial margin; and BTL2, closer to the lake); one at North Star Yedoma (NSY), a well-drained terrestrial hillslope characterized by thermokarst mounds; and one site at Skidmore Pond (SKP), a recently drained (2022) thermokarst pond in hillslope Yedoma near the well-studied Vault Creek Permafrost Tunnel (Schirrmeister et al., 2016; Heslop et al., 2020; Figure 1). Although BTL occupies only < 0.01% of the known permafrost land area it contains widespread sub-aerial taliks situated over Yedoma permafrost, which is estimated to emit between 0.1–5.2 tetragrams of CH_4_ per year, roughly 4% of the entire pan-Arctic wetland budget (Elder et al., 2021).

Using a Talon Coring system (Quantum Machine Works, Whitehorse, Canada) we drilled cores during the winter, when the top ∼1.2 meters of soil was frozen solid. On consecutive days, we drilled on the south boundary of the lake at site BTL1 (A2), then BTL2 (A1), NSY (B1), and SKP (C1) (Figure 1). The depth of each borehole (including depth of the active layer and transition zone) and depth profile are given in Supplementary File S1. We sought to obtain cores at least 5 m long at all talik sites; at the NSY site we were able to reach the permafrost layer ∼6.8 m. After drilling, each core was extracted in half-meter increments, subsampled for plugs, and capped in ABS (acrylonitrile butadiene styrene) pipes obtained from a local hardware supplier. Active layer core increments (frozen solid in the ground) were stored frozen until analysis; cores collected from below the active layer (not fully frozen in the ground) were unfrozen when harvested and stored at 4°C. To enable sub-sampling, frozen core increments were allowed to briefly thaw at room temperature to soften the soil; all subsampled plugs were then stored in air-tight conical tubes and frozen for later microbial and chemical analysis.

### Subsampling soil cores

Sampling equipment was sterilized using 10% bleach/70% ethanol or autoclaved prior to sub-sampling. For metagenomic analysis of microbial populations in the insulated layer below the frozen active layer, plugs were extracted from BTL1, BTL2, SKP, and NSY at 1.5 m depth. Each plug was sampled and sectioned with sterile 5 mL syringes (with the syringe tips excised), then each syringe was used to push a plug into separate 15 mL centrifuge tubes for each depth. Once all plugs are extracted from the core increments, the outer portion of the core increment is discarded, and the inner portion of the core is saved. This sub-sampling approach reduces contamination of the inner core with the outer core microbial fraction. In some cases, soil subsampling was done in an anaerobic chamber to prevent anerobic microbes from exposure to oxygen and other general contaminants. Soil samples were sent to Zymo Research for DNA extraction, V3-V4 16S rRNA gene sequencing, shotgun metagenomics sequencing, and data analysis (Caporaso et al., 2010; Segata et al., 2011; Callahan et al., 2016).

### Elemental chemical analysis

Core samples were sent for elemental chemical analysis at ALS Global (Tucson, Arizona) to measure the percent weight of ash, carbon, hydrogen, nitrogen, and sulfur at various depths for each borehole site core (Supplementary Table S1). Prior to chemical analysis, each soil sample was dried at 60°C. Oxygen was measured using a combustion technique to pyrolyze the sample in an inert atmosphere (helium). Pyrolysis of the soils produces nitrogen, hydrogen, and carbon monoxide when they interact with a nickel-plated carbon catalyst at 1,060°C. These products were separated via a chromatographic column, and the carbon monoxide was analyzed in a thermal conductivity analyzer, providing the oxygen percentage.

### 16S rRNA sequencing and analysis of cores from various depths

For 16S rRNA sequencing, ZymoBIOMICS^®^ -96 MagBead DNA Kit (Zymo Research, Irvine, CA) was used for DNA extraction. Positive controls included ZymoBIOMICS Microbial Community Standard (Zymo Research, Irvine, CA). For 16S rRNA sequencing, DNA libraries were prepared for using the Quick-16S Plus NGS Library Prep Kit (Zymo Research, Irvine, CA). ZymoBIOMIC 16S rRNA gene primers (forward CCTACGGGGNGGCWGCAG, reverse GACTACHVGGGTATCTAATCC) for V3–V4 regions amplification of 16S rRNA genes. The sequencing library was prepared by PCR using real-time PCR thermocycler (QuantStudio 12K Flex, Applied Biosystems) to control cycles and limit PCR chimera formation. Quantification of final PCR products was performed using qPCR fluorescence readings before being pooled together at equal molarity. Pooled libraries were cleaned with DNA Clean & Concentrator kit (Zymo Research, Irvine, CA), and quantified with TapeStation (Agilent Technologies, Santa Clara, CA) and Invitrogen Qubit 1X dsDNA High-Sensitivity Assay Kits (Thermo Fisher Scientific, Waltham, WA). The final library was sequenced on Illumina NextSeq 2000 with a p1 (cat 20075294) reagent kit with 600 cycles. 16S amplicon sequencing was performed with 30% PhiX spike-in. Chimeric sequences were also removed with the Dada2 pipeline (Callahan et al., 2016). Taxonomy assignments were performed using Uclust from Qiime v.1.9.1 (Caporaso et al., 2010). Absolute abundance quantification was performed using qPCR conducted with a standard curve of plasmid DNA with one copy of the 16S gene region prepared in 10-fold serial dilutions (Supplementary Figure S2). Taxonomy was assigned with the Zymo 16S database (Supplementary Figure S3; Supplementary Table S2). Relative taxonomic composition was generated from samples that were sequenced to a 200M read target, and 1M reads were used in downstream processing.

### Shotgun metagenomic library preparation and sequencing of soil core samples

Genomic DNA was extracted from each plug with ZymoBIOMICS^®^ -96 MagBead DNA Kits (Zymo Research, Irvine, CA) and then subjected to shotgun metagenomic sequencing. Illumina^®^ DNA Library Prep Kit (Illumina, San Diego, CA) with up to 500 ng DNA input following the manufacturer’s protocol using unique dual-index 10 bp barcodes with Nextera^®^ adapters (Illumina, San Diego, CA). All libraries were quantified with TapeStation^®^ (Agilent Technologies, Santa Clara, CA) and then pooled to equal abundance. The final pool was quantified using qPCR. The final library was sequenced on an Illumina NovaSeq X (Illumina, San Diego, CA) with 2 × 150 kit (300 cycles). The metagenomic read processing summary more than 2M reads with > 75% reads surviving (Supplementary Table S4).

### Metagenomic bioinformatics and microbial composition analysis

To remove low-quality features, raw sequence reads were trimmed with Trimmomatic-0.33 (Bolger et al., 2014). Quality trimming was conducted via sliding window with 6 bp window size and a quality cutoff of 20; reads with size lower than 70 bp were removed. BBduk (version 39.03) was used to filter the contaminants and adapter sequences from metagenomic raw reads (Singer et al., 2016). The artifact sequences were evaluated and trimmed off by kmer matching (k = 31). The trimmed reads were assembled using MEGAHIT (v 1.2.9) (Li et al., 2015) and the genes were called by Prodigal (v 2.6.2) (Li et al., 2015). The functional profiles were annotated by Eggnog Mapper (v2.1.12) on assembled reads to identify the presence of KOs (KEGG Orthology groups) (Cantalapiedra et al., 2021; Kanehisa et al., 2023). Taxonomy was profiled using Kraken2 (v2.1.3) against the NCBI database (Wood et al., 2019). The resulting taxonomy and abundance information were further analyzed via: (1) alpha- and beta-diversity analyses (Supplementary Figure S2); (2) microbial composition bar plots using QIIME (Caporaso et al., 2012); (3) abundance heatmaps with hierarchical clustering (based on Bray-Curtis dissimilarity); and (4) biomarker discovery with LEfSe (Segata et al., 2011) with default settings (*p* > 0.05 and LDA effect size > 2). Functional profiling was performed on assembled reads using Humann3 (Beghini et al., 2021), including the identification of UniRef gene family and MetaCyc metabolic pathways.

### Gas collection and chemical desorption of VOCs from borehole sites

To capture VOC gasses in the field, we used a stainless-steel gas capture dome with a custom fitting for thermal desorption unit (TDU) tubes. For general VOCs, we used Tenax TA 60/80 from Camsco (Camsco, Houston, TX) placed in-line between a non-polar filter and a calibrated air SKC AirChek pump (SKC, Eighty Four, PA). The non-polar filter was attached between the capture device and the TDU tube to reduce water adsorption on the Tenax TDU. The pump was calibrated with using Mesa Labs 530 + DryCal calibrator (Mesa Labs Inc., Lakewood, Colorado). The capture dome was placed on the borehole immediately after the last core was extracted. At each borehole, 2 L of borehole gas was extracted over 5 min with a calibrated pump flow rate of 0.4 L/min. To provide technical replicates for analysis, triplicate TDU tubes were used to consecutively collect gas from each borehole site. The gas samples were stored at −20°C until 2D-GC-MS analysis.

### 2D-GC-MS instrumental parameters and standards

Samples were thermally desorbed using a Gerstel Thermal Desorption Unit 3.5 + (Gerstel GmbH, Mülheim, Germany) integrated into a gas chromatograph/mass spectrometer system and ramped at 60°C/min from 35°C to either 280°C for Tenax TA sorbent. Desorbed samples were refocused on a Gerstel CIS 4 Cryogenic Inlet, held at -50°C during desorption and ramped at 12°C/s to 300°C to inject the desorbed sample as a single bolus into the gas chromatograph/mass spectrometer system. Analysis of the samples was performed via a LECO Pegasus BT two-dimensional gas chromatograph coupled to a time-of-flight mass spectrometer (2D-GC-MS) (LECO Corp., Michigan, United States). Compound separation was conducted by two analytical gas chromatography columns, with the primary column a 15 m, 0.25 mm ID DB-WAX and secondary column a 2 m, 0.25 mm ID DB-1 (Both Agilent, California, United States), with film thickness of 0.5 μm for the primary column and 1 μm for the secondary column. The Gas Chromatograph analytical program had an initial temperature of 35°C, ramping at 10°C/min to a maximum of 230°C with a 5-minute hold. The secondary column was ramped at the same rate, but with a + 5°C offset from the primary column. Column flow was set to 1 mL/min with an inlet flow split of 50:1. Thermal modulation between columns was set to 7 s with a hot pulse time of 2.10 s. Mass spectra were collected at a 200 spectra/s rate scanning from 20–500 m/z. A 30 kHz extraction frequency and a detector offset of -30 V were used.

### Volatile compound composition and abundance analysis

Chemical identification by 2D-GC-MS detected 1,000 + different chemical features. Top-down quantitative analysis of metabolites in the hit lists was performed using the ChromaTOF TILE software (LECO Corp., St. Joseph, Michigan). TILE divides chromatographs into retention time windows “TILES” and filters these TILES based on the statistical significance (fisher ratio) for each mass identified for facile handling of complex data sets. The size of the TILE accommodates for retention time variation, which can plague other approaches for 2D gas chromatography analysis and is an industry proven tool. In practice, TILE provides the user a qualitative list of VOC chemical classification to search and compare amongst test conditions thus greatly reducing analysis time for “biomarker” hunting. TILEs are then traced to the online NIST23 library for chemical identification. TILE filtering parameters: Tile size D1 (modulations) = 3; Tile size D2 (spectra) = 44; S/N threshold = 50; Samples that must exceed S/N threshold = 2; Mass F-ratios to average = 1; Threshold type = p-value; p-value threshold = 0.05; Minimum masses per tile = 3; Minimum mass = 0; Maximum mass = 1,000; Masses to ignore: = blank.

**FIGURE 3:**
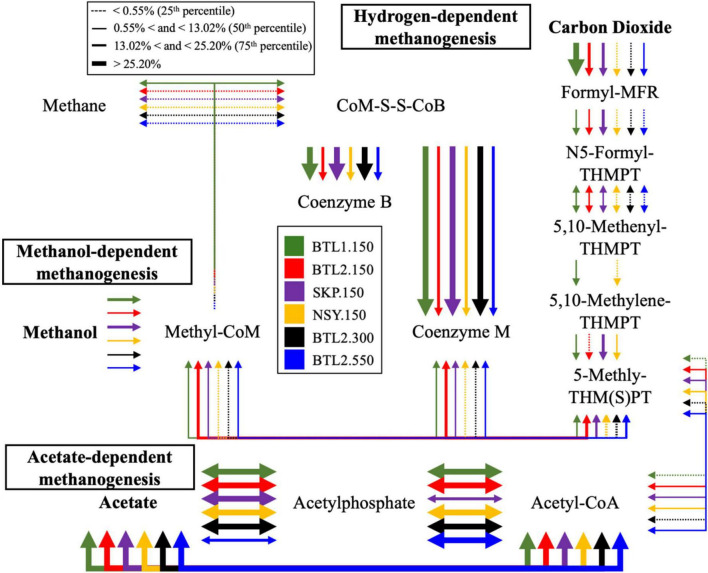
Metabolic pathway analysis was conducted from relative abundance of genes found in the methane metabolic pathway for cores BTL1.150 (green), BTL2.150 (red), SKP.150 (purple), NSY.150 (yellow), BTL2,300 (black), and BTL2.550 (blue). This visualization compares the relative abundance of enzyme genes for each for each pathway leading to methanogenesis including hydrogen-, methanol-, and acetate-dependent methanogenesis. The thickness of the arrows represents the relative percent abundance for each pathway, whereas the thickest arrows indicates that more than 25% of the enzyme genes in these samples where associated with the indicated pathways.

Each individual VOC feature identified by TILES analysis was compared to the NIH PubChem database (accessed June 2024) to check for alternative chemical synonyms. From the > 400 TILES, each VOC feature was then manually compared to the KEGG database to identify related biochemical pathways, genes, and enzymes. We identified 41 unique VOCs that are potentially biogenic in origin and were unique to individual borehole sites in KEGG (Kanehisa et al., 2023), which are indicated in the Biological VOC table in Figure 1. Chemical names were from these data were aligned to a subset of VOCs abundance to gene-hit abundance of enzymes found in related metabolic pathways on KEGG (Figure 3). Triplicate samples were averaged with standard deviations (Supplementary Table S1).

For statistical analysis of VOCs compounds compared across borehole sites a one-way analysis of variance (ANOVA) was performed using GraphPad Prism 10.2.3 (GraphPad Software LLC, Boston, MA) on the triplicate VOC samples found in Supplementary File S3. We performed the ANOVA analysis using the triplicate VOC values obtained from each borehole, averaged them, and compared the variances to determine if the boreholes had significantly different expression of each VOC from Supplementary File S3. Our null hypothesis was that there was no statistical significance of VOC abundance between boreholes. (Supplementary Table S5). As part of our ANOVA summary in Supplementary Table S5 we included the Brown-Forsythe test and Bartlett’s test. Brown–Forsythe test is a statistical test for the equality of group variances based on performing an Analysis of Variance (ANOVA) on a transformation of the response variable. Bartlett’s test tests the hypothesis that our samples have equal variances.

## Results

### Profiling talik sites using geophysical surveys

To better compare corresponding microbial and VOCs signatures between talik sites, we needed to select the sites to provide contrasting examples of talik systems at various stages and magnitudes of thaw. To guide that selection, began by mapping the varying stages of permafrost thaw along hydrological gradients from uplands to lowlands, generating 2D-inversion models from geophysical resistivity surveys using very long frequency-magnetotelluric (VLF-MT) (Elder et al., 2021) for the BTL, NSY, and SKP sites (Figure 1).

Colored contour plots differentiate frozen and unfrozen subsurface features (e.g., aquifers, taliks), allowing us to identify two talik sites at BTL that were only 15 meters apart but exhibited different hydrological and CH_4_-emitting properties. The BTL1 site (A2 in Figure 1) is in the terrace zone of the thermokarst; it was also more saturated than the BTL 2 site and exhibited elevated CH_4_ emission in both the cold and grow season (Figure 1). In contrast, the BTL2 site (A1 in Figure 1) was < 1 m higher in elevation and was a drained-soil site in the littoral zone with a deep talik below 5 m in the soil column; this site had also exhibited natural fluxes up to 5 kg CH_4_ m^–2^ d^–1^ during the cold season (Supplementary Figure S1). The NSY site is a gradually sloped field underlain by thawing, ice-rich Yedoma permafrost that has thermokarst mounds on the surface, which has periodic emissions of CH_4_ after heavy rains. NSY cores were taken near the Eddy-covariance tower site, which has routinely detected CH_4_ emissions year-round. Our NSY 2D-inversion model showed uniform talik and permafrost layers below the frozen active layer. The SKP site was a naturally occurring thermokarst pond near the Skidmore mine that is routinely drained by the landowner to allow the ground to freeze (Figure 1). The SKP 2D-inversion model showed the lowest thaw depth of all our sites near a permafrost tunnel.

Chemical analysis at various depths revealed the highest carbon content at BTL2 at a depth of 200 cm compared to all other core sites (17.49 wt%), hydrogen (1.94 wt%), oxygen (15.27 wt%), and sulfur (0.09 wt%) content (Supplementary Table S1).

### Microbial community structure and diversity

Cores were immediately subsampled at a depth of 150 cm at all sites (BTL1.150, BTL2.150, NSY.150, and SKP.150); the subsampled plugs were transported cold and frozen until we were able to perform 16S and shotgun metagenomic sequencing and analysis.

We used 16S rRNA sequencing to survey microbial compositions at various depths, which indicated microbes involved in methanogenesis including *Methanosarcinales* and *Methanomicrobiales* genera. In total, 708 genera were observed to have known involvement in CH_4_ metabolism at various sites, with the primary CH_4_-producing archaea genera *Methanosarcina* observed at 16.4% relative abundance at BTL1. The microbial community most associated with CH_4_ metabolism were dominated by the genera *Methyloligellaceae, Methyloceanibacte, Hyphomicrobium*, *Bradyrhizobium*, *Pseudomonas*, and *Streptomyces*, which are known to be able to utilize CH_4_ in methanotrophy (Hough et al., 2020).

The most dominant bacterial class detected at NSY and SKP was *Pseudomonadales*, whereas both BTL sites had little to no *Pseudomonadales* with only 3.4% at BTL2 and none at BTL1 where the high seasonal methane flux has been detected. We also detected other notable nitrogen and carbon dioxide fixation species such as *Bradyrhizobium* at BTL2. Likewise, the methylotrophic species *Methyloceanibacter* was 11.2% at BTL1, but also detected at 4.8% for BTL2 at 550 cm depth. The sulfate reducing genera *Desulfobacterota*, *Rhodoferax* and *Pseudomonas* were observed at most sites (Supplementary Figure S3). However, sulfate reducing family *Desulfobacterota* was most abundant with BTL1.150 at 9.1% followed by NSY at 7.3% relative abundance (Supplementary Figure S3; Supplementary Table S3). Additional depths were subsampled from BTL2 at depths 300 cm (BTL2.300) and 550 cm (BTL2.550) and included in our 16S and metagenomic analysis to determine microbiome diversity deeper in the talik at this drained site.

In contrast to metagenomic analysis, 16S sequencing of different depths revealed in total 39 archaeal genera that contained the methyl coenzyme M reductase (*mcrA)* gene; among this population, 4 methanogenic archaea (*Methanosarcina*, *Methanoregula*, *Methanosphaerula*, and *Candidatus Methanogranum*) were observed in our core samples. While *Methanosarcina* was a major methanogenic archaeon at BTL1.150 in the lower active layer, SKP had high relative abundance of *Methanosphaerula* deeper in the soil column around 430 cm SKP.430 (Supplementary Figure S3; Supplementary Table S2). *Actinomycetes* and *Alphaproteobacteria* were present in all microbial communities at all core sites in our metagenomic data.

**FIGURE 4:**
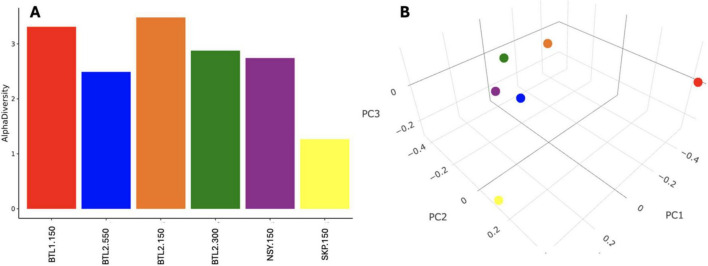
**(A)** Species diversity was quantified from metagenomic datasets using Shannon alpha diversity analysis, which revealed differential microbial richness across core site samples. **(A)** Bray-Curtis dissimilarity analysis generated a 3D principal coordinate analysis (PCoA) at strain level.

**FIGURE 5:**
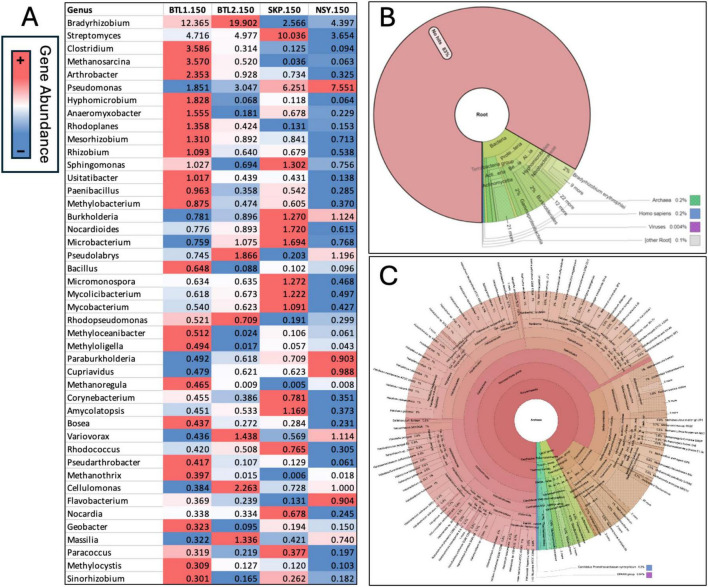
Relative gene abundance was obtained from metagenomic analysis of each core from 150 cm depth below the frozen active layer **(A)** and conditionally formatted and aligned to the highest abundance hits at BTL1 to compare each microbial genera across borehole sites. Krona chart of all metagenomes at BTL1 revealed that 17% of annotated metagenomic sequences were bacteria, archaea, and viruses with the remaining 83% containing genes of unknown function **(B)**. Since BTL1 had the highest number of methanogens, a Krona chart zooming into the archaea fraction at BTL1 was generated, that shows the diversity of methanogenic, halotolerant, and alkaliphilic archaea genera detected from metagenomes **(C)**.

The psychrophile species *Cryosericales* was found at 150 cm for all core sites except for SKP. *Cryosericales* species increased in relative abundance deeper in the soil column at BTL2, moving from 2, 5, and 10.7% at 150, 300, and 550 cm, respectively. Relative abundances of classified methanogens initially reveal that BTL1.150 had the highest amount of *Methanomicrobiales* (3.2%)*, Methanosarcinales* (16.4%)*, Methanotrichales* (0.2%)*, Methanobacteriales* (0.6%), at total of ∼20.4% relative abundance (Supplementary Figure S1). Microbial compositions and species diversity were quantified from metagenomic datasets (Figure 4). Shannon alpha diversity plots were generated from metagenomic analysis and revealed similar microbial richness for BTL1 and BTL2 at 150 cm, whereas the SKP borehole had the lowest level of microbial richness (Figure 4A). Alpha diversity plots also show depth-dependent microbial diversity with lower diversity found deeper in the soil column for BTL2.300 and BTL2.550 compared to BTL2.150. Bray-Curtis dissimilarity analysis of metagenomics microbial analysis revealed that BTL1.150 and SKP.150 had contrasting microbial populations compared to each other as well as compared to all other cores obtained from BTL2 and NSY at similar depths (Figure 4B). Notably, BTL2.150 metagenomic microbial composition had similar populations to the BTL2.300 and BTL2.550 depths. However, we discovered that 17% of the metagenomic sequences matched to bacteria, archaea, and viruses at BTL1 (Figure 5B). The remaining 83% of the metagenomic data contained unclassified genes that represent a major portion of unknown biological activity in these permafrost soil microbiomes, which merits further study to understand functional impacts in biogeochemical cycling.

To understand the absolute number of microbes at 150 cm we focused our analysis on predicted genes and performed analysis microbial composition analysis metagenomes at the genus level. From this analysis we generated a heat map table comparing microbial genus abundance across boreholes at 150 cm depth is displayed with conditional formatting to highlight the microbial abundance across core sites at 150 cm depth (Figure 5A). The BTL1 metagenomes contained a notable diversity of archaea, with many examples of methanogenic and halotolerant species (Figure 5C). These results indicated high abundance of the archaeal *Methanosarcina* family at BTL1 compared to the other cores at 150 cm depths. However, many of the genes and microbes were unclassified with > 80% in the metagenomic data and > 40% in 16S amplicon data that we could not attribute either a predicted function or host.

### Potential metabolic functional genes

Functional gene analysis revealed the potential for CH_4_ production at all sites (Figure 3). BTL1.150 contained genes for hydrogen-, acetate-, and methanol-dependent methanogenesis. Metagenomic analysis of gene counts also showed the highest potential for CH_4_ metabolism at BTL1.150, followed by NSY1.150 (Supplementary File S2). In contrast, gene counts for CH_4_ pathways at SKP1.150 and BTL2.150 were considerably lower. The taxa associated with the central methanol methyl-coenzyme M reductase (EC 2.8.4.1), the enzyme that catalyzes the final step in methanogenesis pathway, was observed to be highest in BTL1.150 (Supplementary File S2), which also had the highest fraction of *Methanosarcina* (16.4%), by more than an order of magnitude, compared to BTL2.150, NSY1.150, and SKP1.150 (< 1.3% *Methanosarcina*). Metagenomic annotations of gene counts for sulfur reduction were present at all sites. In total, 10 archaeal and 681 bacterial genera were associated with sulfate reduction. Among them, 77 potential sulfate-reducing bacterial genera were observed in the various soil core samples including *Desulfobacterota*, *Rhodoferax* and *Pseudomonas*, which have been shown to contain robust sulfate reduction pathways (Chen et al., 2023; Wang et al., 2023; Supplementary Figure S4; Supplementary Table S3). Interestingly, the lowest relative abundance of sulfate reducing bacteria (i.e., *Desulfosporosinus*) was found at SKP for the 430 cm samples, where methanogenic archaea were dominant (Supplementary Figure S3; Supplementary Table S2).

### Characterizing borehole VOCs and their associated biogenic pathways

Our analysis of 2D-GC-MS data revealed biogenic VOC abundance and divergent chemical features between talik boreholes sites. We found > 400 unique VOC features ranging from C2 – C28, with > 40 VOCs linked to microbial and biogenic activity (Supplementary material). Our summary of the relative VOC abundance at each site and conditional formatting reveal distinctive VOC “fingerprints” for each borehole (Supplementary File S3). We were able to identify different classes of VOC compounds (e.g., aromatic hydrocarbons, aldehydes, butenolides, alcohols, ketones, and carbonyls) and derivative compounds (e.g., acetaldehyde, benzene, hexanaldehyde, acetone, tolueneformaldehyde) that have been previously identified as GHGs and linked to global warming (David and Niculescu, 2021).

As documented in Supplementary Table S6, the observed VOC byproducts can also be associated with gene hits for anaerobic styrene degradation, glyoxylate and dicarboxylate metabolism, propanoate metabolism, and volatile fatty acid degradation (e.g., propionate, formate, acetate, and butyrate). In fact, many of the VOCs appeared to be byproducts of enzymatic anaerobic degradation by laccase, oxidoreductase, decarboxylase, and hydrolase enzyme reactions, as inferred from analysis of KEGG pathways (in anaerobic systems acyl lipids are hydrolyzed by lipases.) (Mackie et al., 2008). Notable biogenic VOCs were identified from a manual KEGG database analysis (Supplementary Table S4). VOC abundance profiles were aligned to the genes of degradative enzymes, including cyclohexane to cyclohexane dehydrogenase (EC: 1.3.8.10), isobornyl formate to formylmethanofuran dehydrogenase (EC: 1.2.7.12), acetic acid to acetate kinase (EC: 2.7.2.1), acetophenone to ethylbenzene hydroxylase (EC: 1.17.99.2), styrene to aliphatic nitrilase (EC: 3.5.5.7), and oleic acid (fatty acid degradation product) to catalase (EC: 1.11.1.6) (Figure 3).

## Discussion

### Biogenic VOCs serve as biological indicators of CH_4_-emitting taliks

One of our primary goals was to identify the biological drivers of CH_4_ emissions found at Big Trail Lake during the cold season by comparing VOC profiles at four local thermokarst sites taken from the same transition-layer depth (150 cm) (Supplementary Figure S1). We assumed that permafrost soil microbes control the fate of soil organic carbon (SOC) during various states of thaw, and subsurface VOCs could provide improved resolution of how microbes respond to warming trends. Our preliminary CH_4_ flux measurements identified anomalous CH_4_-hotspots around BTL, which provided a roadmap to investigate microbial transformation of SOC to VOCs under anaerobic conditions in the subsurface (Supplementary Figure S1). We hypothesized that if we measured microbes and their functional metabolisms beneath the frozen active layer, we would be able to identify contrasting biochemical profiles, particularly at sites with different CH_4_ flux measurements.

The results of our proof-of-concept study appear to support our hypothesis. We used 16S rRNA amplicon sequencing to screen microbial populations at the same depth (150 cm) at all four sites, finding that each site hosts unique and diverse microbial communities, including biologically active species related to VOC emissions, such as populations of methane-related microbes (e.g., methanogens, methylotrophs, and methanotrophs). However, all four populations were largely composed of unclassified and uncharacterized taxa and contained many (> 80%) genes of unknown identity and/or function (Figure 5).

**FIGURE 6:**
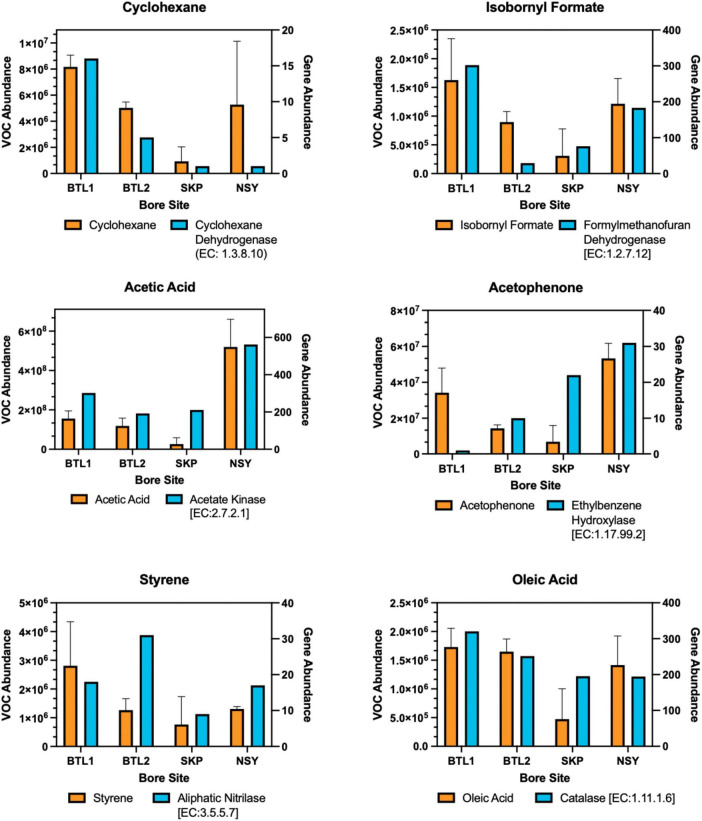
Biogenic VOCs related to anaerobic degradation were aligned to enzyme gene hits for each bore site in a dual y-axis bar plot. The left y-axis shows the mean of triplicate VOC abundance with error bars for one standard deviation from each borehole compared to the right y-axis showing the gene hit abundance of related enzymes obtained from metagenomes for each core site at 150 cm depth. The KEGG enzyme nomenclature number is provided for each enzyme.

Shotgun metagenomics further revealed that the microbiomes associated with each talik site were also distinct from one another, although we observed similarities between metabolic enzyme genes and the specific VOC abundance profiles involved in anaerobic degradation of carbon substrates (e.g., phenolics, aromatics, and organic acids) (Figure 6). Additionally, many VOCs mapped to anaerobic degradation pathways in KEGG (including toluene, ethylbenzene, phenol, styrene, benzoate, and methylnaphthalene), indicating potential linkages between microbial metabolic potential and VOC signatures (Supplementary Table S6). Therefore, VOCs potentially provide novel bioindicators of subsurface biogeochemical changes that can be leveraged for long-term ecosystem monitoring.

### Methanogens, methanotrophs, and microbial communities

Because the active layer is frozen solid during the winter, we hypothesized that samples taken from the protected transition layer (which is slightly warmer due to snowpack thermal insulation) would be most likely to contain microbes that are biologically active during the winter (Holland et al., 2020) and provide the highest likelihood of VOC-to-microbial-composition comparisons. Thus, we again focused our more in-depth metagenomic sequencing on the plugs collected from all four sites at a depth of 150 cm, and at different depths at the same site (guided by the varying depths of the active/transition/permafrost layers). Our particular goal was to identify microbes that accelerate and hinder SOC sequestration, including methanogens, sulfate-reducing genera, and methanotrophs.

For example, by comparing metagenome-resolved microbial composition profiles from different depths at BLT2, we found that extremophilic cold-tolerant species (i.e., psychrophiles), such as *Cryosericales*, increased in abundance with soil depth at that site (Supplementary Figure S4; Supplementary Table S3). These psychrophilic species are particularly important because they are known to play a role in alkane catabolism and carbon cycling; in permafrost soils, psychrophilic species provide low-molecular-C substrates for methanogens (Bowman and Deming, 2014), ensuring that the methanogens can only uptake low-molecular-weight carbon. At BTL1, we also discovered a high diversity of methanogenic species (i.e., *Methanomicrobiales, Methanosarcinales, Methanotrichales, Methanobacteriales)*; however, at SKP and SNY, methanogens were only found deeper in the soil column during (Supplementary Figure S3; Supplementary Table S2). Sulfate reduction is known to reduce methanogenesis potential through several paths (primarily the flow of electrons) (Sela-Adler et al., 2017), so we also looked for sulfur-reducing genera. While *Desulfobacterota* were highest in abundance at the BTL1.150 and NSY.150 sites, but also found in SKP and BTL2 samples, suggesting that …. In contrast, the *Rhodoferax ferrireducens* sulfur-reducing species was not find at either BTL1 but had high abundance in the NSY.150 and BTL2.300 samples, suggesting that….

Methanotrophs (i.e., *Methyloligellaceae*, *Hyphomicrobium, Bradyrhizobium*, *Pseudomonas*, and *Streptomyces*) provide a more direct SOC sink. We detected methanotrophs at higher abundance in the upper 50 cm of the soil column at BTL1, but also present deeper in the soil column at BTL2, SKP, and NSY (thermokarst mounds) (Supplementary Figure S3; Supplementary Table S2). Notably, in the BTL1.150 samples, the dominant methanotroph species were *Methyloligellaceae, Methyloceanibacte, Hyphomicrobium*, but other species that have been reported to stimulate methanotrophy (e.g., *Pseudomonas*) were also present. BTL1 had the highest concentrations of *Methyloceanibacter*, which suggests that C uptake and sequestration is likely responsive to C emissions in these thermokarst systems. In contrast, *Pseudomonas* was the predominate species in the upper 300 cm at SKP and NSY, suggesting possible modes for carbon sequestration from C emissions deeper in the soil column (Veraart et al., 2018). This finding may also explain periodic CH_4_ emissions detected at NSY and SKP sites after heavy rains saturated the thermokarst mounds (Walter Anthony et al., 2024 Manuscript submitted for publication).

Our community structure analysis identified methanotrophic co-occurrence when methanogenic archaea were present in high abundance, which has been previously reported in other soils systems including Arctic soils (Tripathi et al., 2019; Li C. et al., 2021). However, previously, massive field efforts to characterize microbial distributions in permafrost soil found that, as soils thaw, microbial spatial variation is primed and driven by specific biochemical processes (e.g., gas production, metal redox) (Waldrop et al., 2023). Hence, we acknowledge that our limited sample sets cannot draw concrete conclusions of microbial distribution at our talik sites. Still, our observations provide further evidence that methanogenesis can occur lower in the soil column, which has implications for promoting methanotrophy (Singleton et al., 2018) in near-surface soils, thereby capturing and sequestering CH_4_ and other bulk carbon emission before release into the atmosphere.

While we were able to identify of several key methane-related soil microbes and VOC-related metabolic genes, we also discovered that many of the metagenomes we obtained had > 70% unclassified genes. For example, only 17% of the metagenomes from BTL1.150 contained annotated genes for bacteria, archaea, and viruses. The remaining 83% of the metagenomes at BTL1.150 contained unclassified genes (i.e., microbial dark matter), which highlights our limited ability to identify novel extant and extinct genetic traits associated with VOC-related metabolic pathways. Moreover, the diversity of archaea was most pronounced in the BTL1.150 metagenomes, at the site with the highest CH_4_ emissions, which contained many examples of known methanogenic, halophilic, and alkaliphilic genera. Although we couldn’t completely resolve patterns in microbial composition profiles across the cores from various sites and depths, our metagenomic and 16S sequencing of soil depths provided verification that microbes residing in 150–200 cm depths had higher microbial diversity, are potentially active during winter, and could inform how microbial compositions would tie to VOC metabolism at these contrasting talik sites.

### Borehole VOC fingerprints and related anaerobic degradation pathways

**FIGURE 7:**
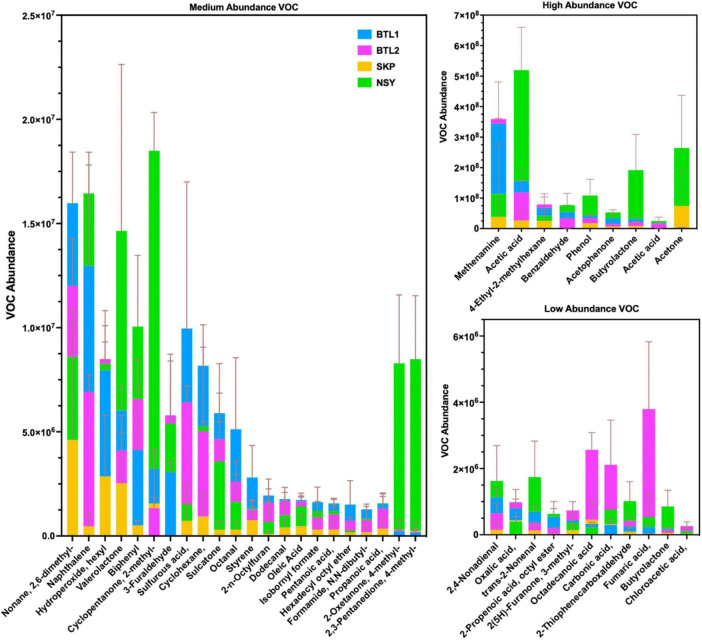
Biogenic VOC abundance from each bore site was collected and analyzed by 2D-GC-MS, and averaged to show medium **(A)**, high **(B)**, and low **(C)** abundance. Bar plots show the mean of triplicate VOC measurements with error bars for one standard deviation. Chemical features with a comma after the name have additional moieties not included in this table but sdf listed in full table VOC features found in Supplementary material.

VOC abundances for each borehole were aligned in a heat map that revealed distinct “fingerprints” for each borehole (Supplementary File S3), highlighting the many VOCs associated with anaerobic degradation pathways of hydrocarbon catabolism (e.g., alkane, toluene, and naphthalene metabolic pathways) and similar enzyme genes associated with hydrocarbon catabolic pathways for propanoate, naphthalene, styrene, and ethylbenzene (Supplementary Table S6). At present, given our limited knowledge of enzyme identity and degradation stages, these anaerobic degradation pathways remain unresolved; however, recent reports indicate that there are four stages of alkane degradation [fumarate addition, C-skeleton re-arrangement, decarboxylation, and beta-oxidation byproducts (Bian et al., 2015)], which are in agreement with the VOCs detected in this study (i.e., fumarate, valerate, butyrate) (Figure 7).

The most abundant VOCs detected from the four talik sites included terminal degradation products consisting mainly of organic acids (e.g., acetic acid, acetone, phenol, acetophenone) (Figure 7B), which likely feed methanogens through the acetate-dependent pathway (Figure 3). We also identified notable biogenic VOCs from a manual KEGG database analysis (Supplementary Table S4), including octanal, acetophenone (Jobst et al., 2010), isothiocyanatocyclohexane (cyclohexane, isothiocyanate) (Hanschen and Schreiner, 2017), phenol (Maurer et al., 2019), furaldehyde (furfural) (Rivard and Grohmann, 1991), furanone (Gómez et al., 2022), styrene (Kanehisa et al., 2023), and benzaldehyde (Huang et al., 2022) (Supplementary Table S4). We also found sulfur-containing VOCs, including sulfur dioxide, isothiocyanatocyclohexane, thiophenecarboxaldehyde, and sulfurous acid (Figure 7). By mapping distinct VOC abundance trends to the gene abundance of associated degradative enzymes (Figure 6), we were able to find remarkable agreement in abundance trends, but also discrepancies that cannot be explained by metagenomics alone, which will require more in-depth functional characterization of enzyme expression. Although many anerobic degradation pathways remain unexplored, our comparative analysis provided a means to understand these talik sites through specific degradation enzymes that correlated to VOC “fingerprints” of boreholes in different thermokarst systems (Supplementary File S3). Although metagenomics doesn’t provide expression or enzyme activities, we were able to infer the presence of functional pathways from gene count abundance. While the ecological role of our measured VOC compounds remains to be elucidated, our results provide insights into the subsurface microbial processes with environmentalsignificance.

### Volatilomics for informing subsurface biokinetics

At each borehole, we identified unique chemical VOC signatures that warrant further study (e.g., via laboratory soil incubations) to statistically define VOC expression patterns and determine transient VOC signatures. In fact, we found many VOCs that are byproducts from various degradation enzymes, including oxidoreductases, lyases, laccases, and carboxylases (Gibson and Harwood, 2002). Although current understanding of microbial anaerobic degradation pathways are limited, recent studies have revealed the scope and complexity of anaerobic biodegradation pathways of n-alkanes in carbon-rich oil reservoirs (Bian et al., 2015). These reports have attempted to define species-specific metabolic pathways using metagenomics and metaproteomics (McGivern et al., 2021); however, VOC “fingerprinting” does not require knowledge of specific enzymatic pathways to identify permafrost states that are likely to facilitate abrupt thaw, talik formation, and thermokarst expansion.

Our alignment of VOCs to degradation enzyme genes provided preliminary evidence that VOC monitoring in Arctic soils is a potentially useful approach to measuring subsurface microbial dynamics over time (Figure 6), because the biochemical alignment can potentially be used to interpret VOC expression to infer subsurface biokinetics, which could provide high-resolution datasets of subsurface SOC transformation, advancing attempts to model the degradation and transformation of SOC. For instance, a recent study used metagenome-assembled genomes to refine the enzyme latch theory and detected similar degradation products (e.g., propionic acid, fumaric acid, and benzaldehyde derivatives), which could be enhanced with VOC “fingerprinting” analysis to further elucidate degradation enzymes and their associated pathways (Wilhelm et al., 2021). Refinement of terrestrial SOC models using VOC-derived biokinetics could also resolve chemical and biological heterogeneity to make soil microbiomes tractable for study in subsurface systems (Jansson and Taş, 2014; Mackelprang et al., 2017; Waldrop et al., 2023).

## Conclusion

The increasing duration of thaw seasons in permafrost ecosystems has created positive feedback loop with gradual and abrupt thaw events, which is deepening and expanding the areas of year-round unfrozen ground (i.e., taliks) (Walter Anthony et al., 2018; Turetsky et al., 2020; Walter Anthony et al., 2021). The lack of high-resolution data in subsurface Arctic terrestrial ecosystems limits our ability to understand the subsurface biogeochemical drivers which drive the rate and magnitude of environmentally triggered SOC turnover (Bradford et al., 2016; Tao et al., 2024). Our study demonstrates a proof-of-concept that VOCs can potentially serve as bioindicators of subsurface biogeochemical processes, providing high-resolution data and broad-scale measurements that can be used to characterize biological roles in the thermokarst–permafrost continuum. This study also establishes methods and approaches for effectively capturing VOCs during winter seasons that could allow for more accurate measurements of subsurface microbial rates of C conversion. Although further validation is required to determine the full range of biogenic VOC “fingerprints” that represent subsurface dynamics and indicate the onset of thaw events (i.e., precursors of gradual and abrupt thaw), these results provide an identification-based approach to track and understand permafrost thaw states responsible for CH_4_ emissions during all seasons in Alaska. Future work should integrate measurements of VOC “fingerprints” derived from laboratory soil incubations at different thaw states to understand the metabolic drivers that shift microbial C cycling during and allocation in arctic soils and translated to other soil systems.

## Data Availability

TILES analysis of untargeted volatilomics using 2D-GC-MS quantification can be found in the Supplementary Data Sheet 1. Metagenome 16S rRNA gene and amplicon sequencing data are available in NCBI under BioProject accession no. PRJNA1216023 (for biosample accession numbers, see Supplementary Data Sheet 2).
