# Supplementary material for: Bioindicator “fingerprints” of methane-emitting thermokarst features in Alaskan soils
Source: Front Microbiol. 2025 Feb 21;15:1462941. doi: 10.3389/fmicb.2024.1462941 (PMC11885255; doi:10.3389/fmicb.2024.1462941)
Supplement: Supplementary file 3 [file Data_Sheet_3.pdf]

Frontiers Supplemental Figures

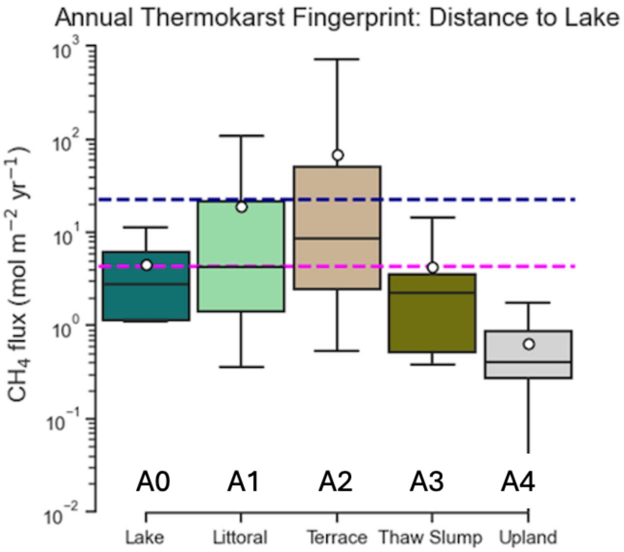

ID	Season	Land Position	Min	Mean	Max
1	Cold	Littoral	0.17	2.15	8.01
2	Cold	Wet Terrace	0.30	2.58	9.70
3	Cold	Dry Terrace	0.08	1.09	2.99
4	Cold	Terrace Scarp	0.25	5.01	36.20
5	Cold	Perm. Bluff	0.13	3.30	10.79
6	Cold	Forest Upland	0.00	0.12	0.76
7	Growing	Littoral	0.36	18.85	107.5
8	Growing	Wet Terrace	2.99	48.13	155.4
9	Growing	Dry Terrace	0.53	1.72	3.78
10	Growing	Terrace Scarp	1.31	114.6	713.2
11	Growing	Permafrost Bluff	0.38	4.25	14.39
12	Growing	Forest Upland	0.00	0.64	1.75

Supplemental Figure 1 Annual methane flux averages were collected during cold and grow seasons at BTL thermokarst lake. The bar graph shows methane flux moving outward from the center of BTL with lake (A0), littoral (A1), terrace (A2), thaw slump (A3), and upland (A4) areas. The table lists the methane flux measured during cold and growing seasons for each lake feature that includes the upper and lower limits along with the mean methane flux for each lake feature in Figure 2.

Sample	Ash (wt%)	Carbon (wt%)	Hydrogen (wt%)	Nitrogen (wt%)	Oxygen (wt%)	Sulfur (wt%)
**BTL1.150**	88.91	3.33	0.73	0.15	6.81	0.07
**BTL1.400**	92.82	2.42	0.21	<0.10	4.51	0.04
**BTL2.200**	64.65	17.49	1.94	0.56	15.27	0.09
**BTL2.400**	95.29	1.43	<0.20	<0.10	3.25	0.03
**SKP.150**	94.16	2.02	<0.20	<0.10	3.775	0.07
**SKP.430**	81.34	7.54	0.55	0.51	9.98	0.08
**NSY.150**	93.92	2.21	<0.20	<0.10	3.82	0.05
**NSY.400**	94.9	1.62	<0.20	<0.10	3.42	0.06
**NSY.700**	95.46	1.64	<0.20	<0.10	2.85	0.05

Supplemental Table 1 Elemental analysis of soils from various core sites BTL, NSY, and SKP at depths of 150, 200, 400, and 700 are provided by weight percent for ash, carbon, hydrogen, nitrogen, oxygen, and sulfur content. Sample identifiers represent the following sites and depths. BTL1.150=BTL1 at 150cm; BTL1.400=BTL1 at 400cm,; BTL2.200=BTL2 at 200cm; SKP.150=SKP at 150cm; SKP.430=SKP at 430cm; NSY.150=NSY at 150cm; NSY.400=NSY at 400cm; NSY.700=NSY at 700cm. BTL2.200 contained the highest amounts of every chemical feature with C at 17.59%.

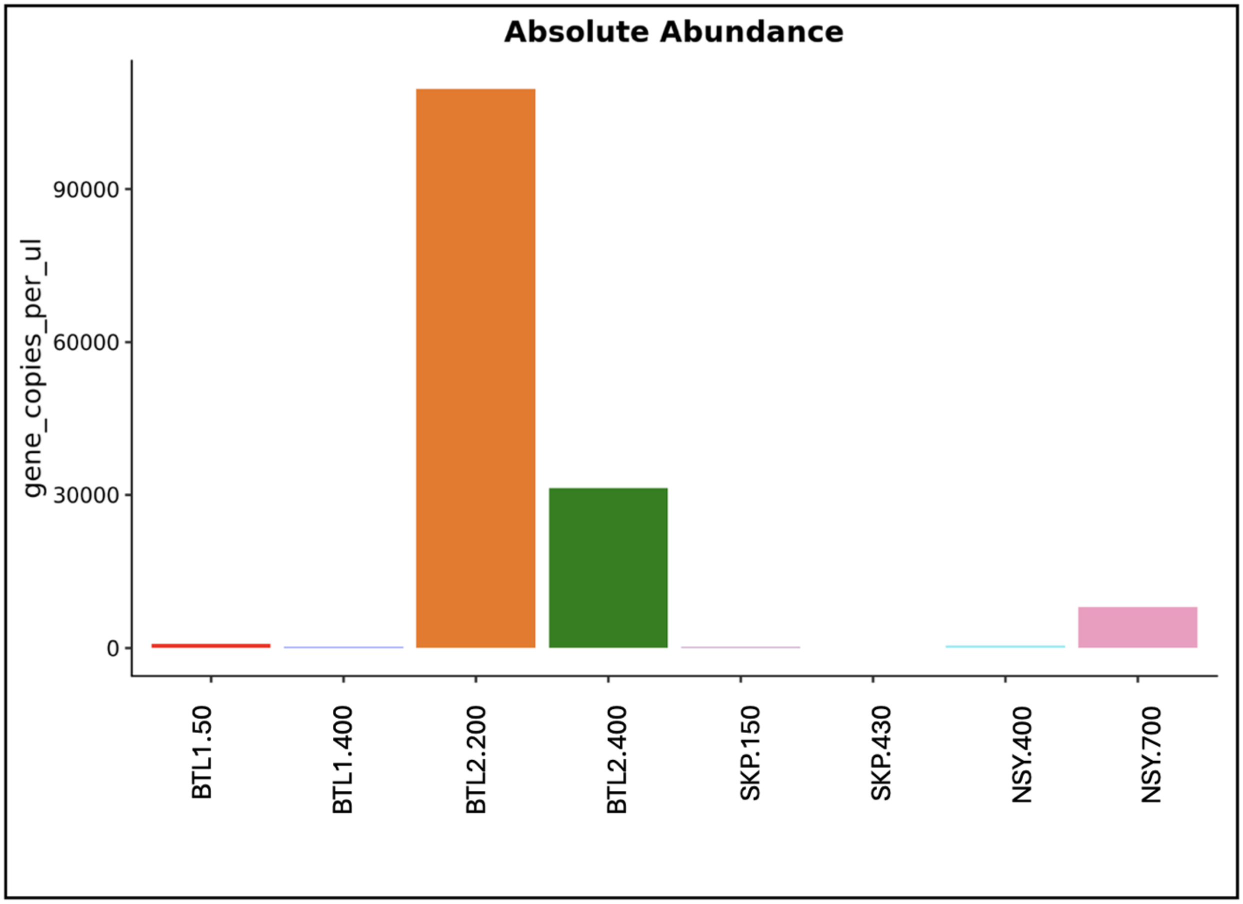

Sample	Ct	Gene Copies per µL	Genome copies per µL	DNA (ng/µL)
**BTL1.50**	29.5	820	205	0.0010425
**BTL1.400**	32.21	126	32	0.0001627
**BTL2.200**	22.49	109706	27427	0.139476
**BTL2.400**	24.54	31386	7847	0.0399048
**SKP.150**	30.17	516	129	0.000656
**SKP.430**	26.56	8030	2008	0.0102114
**NSY.400**	33.29	137	34	0.0001729
**NSY.700**	36.45	12	3	0.0000153

Supplemental Figure 2 The histogram showing the number of gene copies per microliter in each sample was generated to determine the absolute abundance of bacterial (16S) DNA measured for each sample. The table shows the gene copies per microliter of DNA along with genome copies per microliter and DNA concentration for each sample.

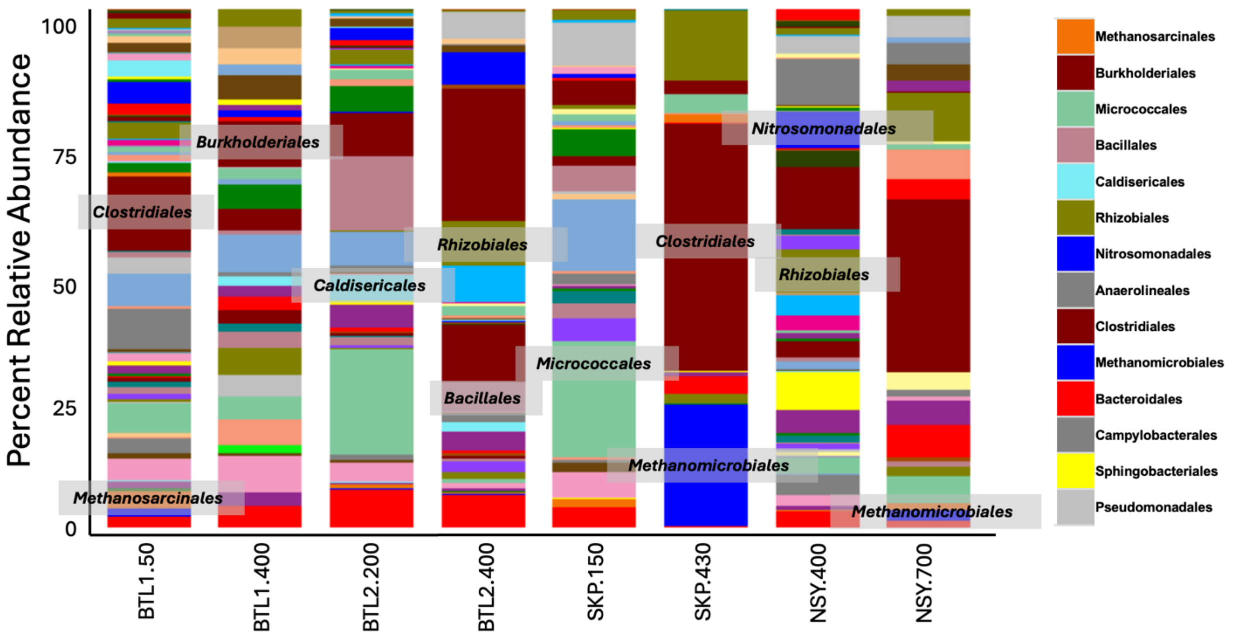

Supplemental Figure 3 Bar plots representing relative abundance profiles of microbial composition at the order level from 16S sequencing are featured in supplemental table 2.

Supplemental Table 2 Table representing relative abundance profiles of microbial composition at the class level from 16S sequencing.

		Total	BLT1.150	BTL1.400	BTL2.200	BTL2.400	SKP.150	SKP.430	NSY.400	NSY.700
Legend	Taxonomy	%	%	%	%	%	%	%	%	%
	None;Other;Other;Other	3.5%	2.0%	4.2%	7.3%	6.2%	3.9%	0.2%	3.0%	1.2%
	k__Archaea;p__Euryarchaeota;c__Methanomicrobia;o__Methanomicrobiales	3.5%	1.7%	0.0%	0.1%	0.2%	0.0%	23.5%	0.0%	2.2%
	k__Archaea;p__Euryarchaeota;c__Methanomicrobia;o__Methanosarcinales	1.0%	3.4%	0.0%	0.8%	0.4%	1.7%	0.0%	0.3%	1.2%
	k__Archaea;p__Euryarchaeota;c__Thermoplasmata;o__Thermoplasmatales	0.1%	0.6%	0.0%	0.0%	0.1%	0.0%	0.3%	0.1%	0.0%
	k__Archaea;p__Miscellaneous Crenarchaeotic Group;c__NA;o__NA	0.6%	1.1%	2.5%	0.4%	0.6%	0.0%	0.0%	0.6%	0.0%
	k__Archaea;p__Thaumarchaeota;c__NA;o__NA	0.0%	0.0%	0.0%	0.0%	0.0%	0.1%	0.0%	0.0%	0.0%
	k__Bacteria;p__Acidobacteria;c__Acidobacteria;o__Acidobacteriales	0.1%	0.3%	0.0%	0.1%	0.0%	0.0%	0.0%	0.0%	0.0%
	k__Bacteria;p__Acidobacteria;c__Acidobacteria;o__NA	2.9%	4.2%	7.1%	3.7%	0.9%	5.1%	0.0%	2.2%	0.0%
	k__Bacteria;p__Acidobacteria;c__Holophagae;o__Holophagales	0.0%	0.0%	0.0%	0.0%	0.0%	0.0%	0.0%	0.0%	0.0%
	k__Bacteria;p__Acidobacteria;c__Holophagae;o__NA	0.5%	1.1%	0.6%	0.5%	0.0%	1.6%	0.0%	0.0%	0.0%
	k__Bacteria;p__Actinobacteria;c__Acidimicrobiia;o__Acidimicrobiales	1.1%	2.8%	0.0%	1.0%	0.0%	0.7%	0.0%	4.0%	0.0%
	k__Bacteria;p__Actinobacteria;c__Actinobacteria;o__Bifidobacteriales	0.2%	0.0%	1.5%	0.0%	0.0%	0.0%	0.0%	0.0%	0.0%
	k__Bacteria;p__Actinobacteria;c__Actinobacteria;o__Corynebacteriales	0.7%	0.4%	4.9%	0.1%	0.0%	0.3%	0.0%	0.0%	0.0%
	k__Bacteria;p__Actinobacteria;c__Actinobacteria;o__Frankiales	0.0%	0.0%	0.0%	0.1%	0.0%	0.0%	0.0%	0.1%	0.0%
	k__Bacteria;p__Actinobacteria;c__Actinobacteria;o__Kineosporiales	0.1%	0.8%	0.0%	0.0%	0.0%	0.0%	0.0%	0.0%	0.0%
	k__Bacteria;p__Actinobacteria;c__Actinobacteria;o__Micrococcales	7.8%	5.6%	4.3%	20.2%	0.8%	22.6%	0.0%	3.2%	5.4%
	k__Bacteria;p__Actinobacteria;c__Actinobacteria;o__Micromonosporales	0.0%	0.0%	0.0%	0.0%	0.0%	0.0%	0.0%	0.2%	0.0%
	k__Bacteria;p__Actinobacteria;c__Actinobacteria;o__NA	0.1%	0.0%	0.0%	0.0%	0.0%	0.0%	0.0%	1.0%	0.0%
	k__Bacteria;p__Actinobacteria;c__Actinobacteria;o__Propionibacteriales	0.6%	0.2%	4.2%	0.0%	0.0%	0.0%	0.0%	0.5%	0.0%
	k__Bacteria;p__Actinobacteria;c__Actinobacteria;o__Pseudonocardiales	0.0%	0.0%	0.0%	0.0%	0.0%	0.0%	0.0%	0.0%	0.0%
	k__Bacteria;p__Actinobacteria;c__Actinobacteria;o__Streptomycetales	0.0%	0.0%	0.0%	0.0%	0.1%	0.0%	0.0%	0.1%	0.0%
	k__Bacteria;p__Actinobacteria;c__Actinobacteria;o__Streptosporangiales	0.0%	0.0%	0.0%	0.0%	0.0%	0.0%	0.0%	0.0%	0.0%
	k__Bacteria;p__Actinobacteria;c__Coriobacteriia;o__Coriobacteriales	1.4%	0.7%	5.2%	0.2%	1.3%	0.0%	1.9%	0.0%	1.6%
	k__Bacteria;p__Actinobacteria;c__NA;o__NA	1.1%	1.0%	0.0%	0.6%	1.9%	4.2%	0.0%	1.0%	0.2%
	k__Bacteria;p__Actinobacteria;c__Thermoleophilia;o__Gaiellales	1.4%	1.4%	3.2%	1.6%	0.5%	3.1%	0.0%	0.1%	1.1%

k__Bacteria;p__Actinobacteria;c__Thermoleophilia;o__Solirubrobacterales	0.8%	0.9%	1.4%	0.2%	0.0%	2.4%	0.0%	1.3%	0.0%
k__Bacteria;p__Aminicenantes;c__NA;o__NA	0.6%	0.8%	2.7%	0.4%	0.5%	0.0%	0.0%	0.0%	0.0%
k__Bacteria;p__Armatimonadetes;c__NA;o__NA	0.0%	0.1%	0.0%	0.0%	0.0%	0.0%	0.0%	0.0%	0.0%
k__Bacteria;p__Atribacteria;c__NA;o__NA	0.2%	0.0%	0.0%	0.3%	0.5%	0.0%	0.0%	0.0%	0.7%
k__Bacteria;p__Bacteroidetes;c__Bacteroidia;o__Bacteroidales	1.7%	0.3%	2.6%	0.8%	0.5%	0.0%	3.3%	0.1%	6.4%
k__Bacteria;p__Bacteroidetes;c__Bacteroidia;o__NA	0.0%	0.0%	0.0%	0.0%	0.1%	0.0%	0.0%	0.0%	0.0%
k__Bacteria;p__Bacteroidetes;c__Cytophagia;o__Cytophagales	0.0%	0.0%	0.0%	0.0%	0.0%	0.0%	0.0%	0.1%	0.0%
k__Bacteria;p__Bacteroidetes;c__Flavobacteriia;o__Flavobacteriales	0.1%	0.3%	0.0%	0.0%	0.0%	0.5%	0.0%	0.3%	0.0%
k__Bacteria;p__Bacteroidetes;c__NA;o__NA	2.8%	1.7%	2.1%	4.5%	3.7%	0.4%	0.7%	4.6%	4.7%
k__Bacteria;p__Bacteroidetes;c__Sphingobacteriia;o__Sphingobacteriales	1.1%	0.9%	0.0%	0.7%	0.1%	0.0%	0.1%	7.2%	0.0%
k__Bacteria;p__Caldiserica;c__Caldisericia;o__Caldisericales	1.1%	0.0%	1.8%	4.9%	1.7%	0.0%	0.0%	0.2%	0.0%
k__Bacteria;p__Chlamydiae;c__Chlamydiae;o__Chlamydiales	0.4%	1.5%	0.0%	0.4%	0.0%	0.4%	0.0%	0.0%	0.8%
k__Bacteria;p__Chlorobi;c__Chlorobia;o__Chlorobiales	0.0%	0.2%	0.0%	0.0%	0.0%	0.0%	0.0%	0.1%	0.0%
k__Bacteria;p__Chlorobi;c__Ignavibacteria;o__Ignavibacteriales	0.1%	0.5%	0.0%	0.2%	0.0%	0.0%	0.0%	0.3%	0.0%
k__Bacteria;p__Chloroflexi;c__Anaerolineae;o__Anaerolineales	1.9%	7.8%	0.7%	1.3%	1.7%	2.1%	0.0%	0.0%	1.2%
k__Bacteria;p__Chloroflexi;c__Dehalococcoidia;o__Dehalococcoidales	0.0%	0.0%	0.0%	0.0%	0.0%	0.0%	0.0%	0.0%	0.0%
k__Bacteria;p__Chloroflexi;c__Dehalococcoidia;o__NA	0.2%	0.5%	0.0%	0.1%	0.3%	0.4%	0.0%	0.0%	0.0%
k__Bacteria;p__Chloroflexi;c__NA;o__NA	4.4%	6.2%	7.4%	6.4%	0.2%	13.9%	0.0%	1.4%	0.0%
k__Bacteria;p__Chloroflexi;c__Thermomicrobia;o__NA	0.1%	0.0%	0.0%	0.0%	0.0%	0.9%	0.0%	0.0%	0.0%
k__Bacteria;p__Chloroflexi;c__Thermomicrobia;o__Sphaerobacterales	0.0%	0.0%	0.0%	0.0%	0.0%	0.0%	0.0%	0.0%	0.0%
k__Bacteria;p__Cloacimonetes;c__NA;o__NA	0.0%	0.0%	0.0%	0.0%	0.0%	0.0%	0.0%	0.0%	0.0%
k__Bacteria;p__Cyanobacteria;c__Chloroplast;o__Monomorphina pseudonordstedti	0.4%	0.0%	0.0%	0.0%	0.0%	0.0%	0.0%	0.0%	3.3%
k__Bacteria;p__Cyanobacteria;c__Chloroplast;o__NA	0.5%	3.1%	0.0%	0.0%	0.0%	0.6%	0.0%	0.0%	0.0%
k__Bacteria;p__Cyanobacteria;c__Melainabacteria;o__Gastranaerophilales	0.0%	0.0%	0.0%	0.0%	0.0%	0.0%	0.0%	0.0%	0.0%
k__Bacteria;p__Cyanobacteria;c__NA;o__NA	0.0%	0.0%	0.0%	0.0%	0.0%	0.0%	0.0%	0.0%	0.0%
k__Bacteria;p__Deinococcus-Thermus;c__Deinococci;o__NA	0.0%	0.0%	0.0%	0.0%	0.1%	0.0%	0.0%	0.0%	0.0%
k__Bacteria;p__Elusimicrobia;c__Elusimicrobia;o__NA	0.0%	0.2%	0.0%	0.2%	0.0%	0.0%	0.0%	0.0%	0.0%
k__Bacteria;p__Fibrobacteres;c__Fibrobacteria;o__Fibrobacterales	0.0%	0.0%	0.0%	0.0%	0.0%	0.0%	0.0%	0.1%	0.0%
k__Bacteria;p__Firmicutes;c__Bacilli;o__Bacillales	3.4%	1.0%	0.8%	14.4%	5.5%	4.9%	0.0%	0.7%	0.0%

	k__Bacteria;p__Firmicutes;c__Bacilli;o__Lactobacillales	0.0%	0.2%	0.0%	0.0%	0.0%	0.0%	0.0%	0.0%	0.2%
	k__Bacteria;p__Firmicutes;c__Clostridia;o__Clostridiales	15.5%	14.4%	4.2%	8.3%	10.9%	1.8%	47.7%	3.3%	33.2%
	k__Bacteria;p__Firmicutes;c__Clostridia;o__NA	0.0%	0.0%	0.0%	0.0%	0.2%	0.0%	0.0%	0.0%	0.0%
	k__Bacteria;p__Firmicutes;c__Clostridia;o__Thermoanaerobacterales	0.0%	0.0%	0.0%	0.0%	0.3%	0.0%	0.0%	0.0%	0.0%
	k__Bacteria;p__Firmicutes;c__Erysipelotrichia;o__Erysipelotrichales	0.5%	0.0%	0.0%	0.0%	0.1%	0.0%	0.3%	0.0%	3.9%
	k__Bacteria;p__Firmicutes;c__NA;o__NA	0.0%	0.0%	0.0%	0.2%	0.1%	0.0%	0.0%	0.0%	0.0%
	k__Bacteria;p__Firmicutes;c__Negativicutes;o__Selenomonadales	0.4%	0.9%	0.0%	0.1%	0.2%	0.0%	1.7%	0.0%	0.0%
	k__Bacteria;p__Gemmatimonadetes;c__Gemmatimonadetes;o__Gemmatimonadales	2.0%	1.6%	4.5%	4.8%	0.0%	5.1%	0.0%	0.3%	0.0%
	k__Bacteria;p__Gemmatimonadetes;c__Gemmatimonadetes;o__NA	0.2%	0.0%	0.0%	0.0%	0.0%	0.1%	0.0%	1.2%	0.0%
	k__Bacteria;p__Gracilibacteria;c__NA;o__NA	0.0%	0.0%	0.0%	0.0%	0.0%	0.3%	0.0%	0.0%	0.0%
	k__Bacteria;p__Hydrogenedentes;c__NA;o__NA	0.1%	0.4%	0.0%	0.1%	0.1%	0.0%	0.0%	0.0%	0.0%
	k__Bacteria;p__Ignavibacteriae;c__Ignavibacteria;o__Ignavibacteriales	0.0%	0.2%	0.0%	0.0%	0.0%	0.0%	0.0%	0.0%	0.0%
	k__Bacteria;p__Latescibacteria;c__NA;o__NA	0.0%	0.0%	0.0%	0.0%	0.0%	0.0%	0.0%	0.0%	0.0%
	k__Bacteria;p__Lentisphaerae;c__NA;o__NA	0.0%	0.0%	0.0%	0.1%	0.2%	0.0%	0.0%	0.0%	0.0%
	k__Bacteria;p__Lentisphaerae;c__Oligosphaeria;o__Oligosphaerales	0.0%	0.0%	0.0%	0.0%	0.1%	0.0%	0.0%	0.0%	0.0%
	k__Bacteria;p__Microgenomates;c__NA;o__NA	0.0%	0.0%	0.0%	0.0%	0.0%	0.0%	0.0%	0.0%	0.0%
	k__Bacteria;p__NA;c__NA;o__NA	1.2%	1.1%	0.0%	1.1%	0.6%	0.5%	0.3%	0.0%	5.7%
	k__Bacteria;p__Nitrospirae;c__Nitrospira;o__Nitrospirales	0.3%	0.5%	1.1%	0.0%	0.0%	0.7%	0.0%	0.0%	0.0%
	k__Bacteria;p__Omnitrophica;c__NA;o__NA	0.0%	0.0%	0.0%	0.0%	0.0%	0.0%	0.0%	0.0%	0.0%
	k__Bacteria;p__Parcubacteria;c__NA;o__NA	1.7%	1.0%	2.2%	1.9%	1.8%	1.4%	3.5%	0.5%	1.1%
	k__Bacteria;p__Planctomycetes;c__NA;o__NA	0.0%	0.1%	0.0%	0.0%	0.0%	0.0%	0.0%	0.0%	0.0%
	k__Bacteria;p__Planctomycetes;c__Phycisphaerae;o__NA	0.3%	0.0%	0.0%	0.2%	0.3%	0.9%	0.0%	0.1%	0.5%
	k__Bacteria;p__Planctomycetes;c__Phycisphaerae;o__Phycisphaerales	0.1%	0.2%	0.2%	0.1%	0.1%	0.0%	0.0%	0.0%	0.0%
	k__Bacteria;p__Planctomycetes;c__Planctomycetacia;o__Planctomycetales	0.6%	0.9%	0.0%	0.5%	0.3%	0.1%	0.0%	2.8%	0.0%
	k__Bacteria;p__Proteobacteria;c__Alphaproteobacteria;o__Caulobacterales	1.4%	0.2%	0.0%	0.3%	7.0%	0.0%	0.0%	4.0%	0.0%
	k__Bacteria;p__Proteobacteria;c__Alphaproteobacteria;o__NA	0.0%	0.0%	0.0%	0.0%	0.0%	0.0%	0.0%	0.3%	0.0%
	k__Bacteria;p__Proteobacteria;c__Alphaproteobacteria;o__Rhizobiales	4.1%	3.2%	0.0%	2.9%	8.4%	0.9%	0.0%	8.5%	9.2%
	k__Bacteria;p__Proteobacteria;c__Alphaproteobacteria;o__Rhodospirillales	0.3%	0.0%	0.0%	0.2%	0.0%	0.0%	0.0%	2.4%	0.0%
	k__Bacteria;p__Proteobacteria;c__Alphaproteobacteria;o__Rickettsiales	0.1%	0.1%	0.0%	0.0%	0.0%	0.0%	0.0%	0.4%	0.0%

	k__Bacteria;p__Proteobacteria;c__Alphaproteobacteria;o__Sphingomonadales	0.2%	0.2%	0.0%	0.1%	0.1%	0.0%	0.0%	1.0%	0.0%
	k__Bacteria;p__Proteobacteria;c__Betaproteobacteria;o__Burkholderiales	6.9%	0.8%	8.9%	0.2%	25.6%	4.7%	2.6%	12.0%	0.2%
	k__Bacteria;p__Proteobacteria;c__Betaproteobacteria;o__Hydrogenophilales	0.5%	0.3%	0.0%	0.1%	0.0%	0.0%	0.0%	3.2%	0.0%
	k__Bacteria;p__Proteobacteria;c__Betaproteobacteria;o__Methylophilales	0.1%	0.2%	0.0%	0.1%	0.6%	0.0%	0.0%	0.1%	0.0%
	k__Bacteria;p__Proteobacteria;c__Betaproteobacteria;o__NA	0.6%	2.1%	0.8%	1.0%	0.0%	0.4%	0.0%	0.5%	0.0%
	k__Bacteria;p__Proteobacteria;c__Betaproteobacteria;o__Nitrosomonadales	2.7%	4.1%	1.2%	2.4%	6.4%	0.8%	0.0%	6.9%	0.0%
	k__Bacteria;p__Proteobacteria;c__Betaproteobacteria;o__Rhodocyclales	0.0%	0.2%	0.0%	0.0%	0.0%	0.0%	0.0%	0.1%	0.0%
	k__Bacteria;p__Proteobacteria;c__Deltaproteobacteria;o__Bdellovibrionales	0.1%	0.3%	0.0%	0.0%	0.0%	0.0%	0.0%	0.8%	0.0%
	k__Bacteria;p__Proteobacteria;c__Deltaproteobacteria;o__Desulfobacterales	0.4%	0.2%	1.0%	0.0%	0.0%	0.0%	0.0%	0.0%	2.3%
	k__Bacteria;p__Proteobacteria;c__Deltaproteobacteria;o__Desulfuromonadales	0.3%	0.6%	1.2%	0.0%	0.0%	0.0%	0.0%	0.2%	0.0%
	k__Bacteria;p__Proteobacteria;c__Deltaproteobacteria;o__Myxococcales	0.4%	3.1%	0.0%	0.1%	0.0%	0.0%	0.0%	0.0%	0.0%
	k__Bacteria;p__Proteobacteria;c__Deltaproteobacteria;o__NA	0.3%	1.3%	0.0%	0.1%	0.1%	1.3%	0.0%	0.0%	0.0%
	k__Bacteria;p__Proteobacteria;c__Deltaproteobacteria;o__Oligoflexales	0.0%	0.3%	0.0%	0.0%	0.0%	0.0%	0.0%	0.0%	0.0%
	k__Bacteria;p__Proteobacteria;c__Deltaproteobacteria;o__Syntrophobacterales	1.5%	1.7%	4.6%	1.6%	1.3%	0.0%	0.0%	0.1%	3.1%
	k__Bacteria;p__Proteobacteria;c__Epsilonproteobacteria;o__Campylobacterales	1.6%	0.0%	0.0%	0.0%	0.1%	0.0%	0.0%	9.0%	4.1%
	k__Bacteria;p__Proteobacteria;c__Epsilonproteobacteria;o__NA	0.0%	0.0%	0.0%	0.0%	0.0%	0.0%	0.0%	0.0%	0.0%
	k__Bacteria;p__Proteobacteria;c__Gammaproteobacteria;o__Cellvibrionales	0.0%	0.0%	0.0%	0.0%	0.0%	0.0%	0.0%	0.1%	0.0%
	k__Bacteria;p__Proteobacteria;c__Gammaproteobacteria;o__Enterobacteriales	0.4%	0.0%	2.2%	0.0%	0.0%	0.0%	0.0%	0.0%	1.1%
	k__Bacteria;p__Proteobacteria;c__Gammaproteobacteria;o__Legionellales	0.8%	1.3%	3.1%	0.1%	1.2%	0.4%	0.0%	0.2%	0.0%
	k__Bacteria;p__Proteobacteria;c__Gammaproteobacteria;o__Methylococcales	0.1%	0.7%	0.0%	0.0%	0.0%	0.0%	0.0%	0.0%	0.0%
	k__Bacteria;p__Proteobacteria;c__Gammaproteobacteria;o__NA	0.1%	0.4%	0.0%	0.0%	0.0%	0.0%	0.0%	0.0%	0.0%
	k__Bacteria;p__Proteobacteria;c__Gammaproteobacteria;o__Oceanospirillales	0.1%	0.0%	0.0%	0.0%	0.0%	0.0%	0.0%	0.6%	0.0%
	k__Bacteria;p__Proteobacteria;c__Gammaproteobacteria;o__Pseudomonadales	2.7%	0.2%	0.0%	0.3%	5.1%	8.2%	0.0%	3.5%	4.2%
	k__Bacteria;p__Proteobacteria;c__Gammaproteobacteria;o__Thiotrichales	0.0%	0.0%	0.0%	0.0%	0.0%	0.0%	0.0%	0.0%	0.0%

	k__Bacteria;p__Proteobacteria;c__Gammaproteobacteria;o__Xanthomonadales	0.2%	0.3%	0.0%	0.6%	0.0%	0.7%	0.0%	0.2%	0.0%
	k__Bacteria;p__Proteobacteria;c__NA;o__NA	0.5%	0.0%	4.1%	0.0%	0.0%	0.0%	0.0%	0.0%	0.0%
	k__Bacteria;p__Saccharibacteria;c__NA;o__NA	2.9%	1.7%	3.5%	0.4%	0.2%	1.6%	13.7%	1.3%	1.2%
	k__Bacteria;p__Spirochaetae;c__Spirochaetes;o__Spirochaetales	0.0%	0.0%	0.0%	0.1%	0.0%	0.0%	0.0%	0.0%	0.0%
	k__Bacteria;p__Synergistetes;c__Synergistia;o__Synergistales	0.0%	0.0%	0.0%	0.0%	0.0%	0.0%	0.1%	0.0%	0.0%
	k__Bacteria;p__Tenericutes;c__Mollicutes;o__NA	0.0%	0.1%	0.0%	0.0%	0.0%	0.0%	0.1%	0.0%	0.0%
	k__Bacteria;p__Verrucomicrobia;c__NA;o__NA	0.2%	0.9%	0.0%	0.1%	0.2%	0.0%	0.0%	0.1%	0.0%
	k__Bacteria;p__Verrucomicrobia;c__Opitutae;o__Opitutales	0.2%	0.6%	0.0%	0.0%	0.0%	0.0%	0.0%	1.2%	0.0%
	k__Bacteria;p__Verrucomicrobia;c__Spartobacteria;o__Chthoniobacterales	0.1%	0.3%	0.0%	0.1%	0.1%	0.3%	0.0%	0.2%	0.0%
	k__Bacteria;p__Verrucomicrobia;c__Verrucomicrobiae;o__Verrucomicrobiales	0.3%	0.0%	0.0%	0.0%	0.0%	0.0%	0.0%	2.0%	0.0%

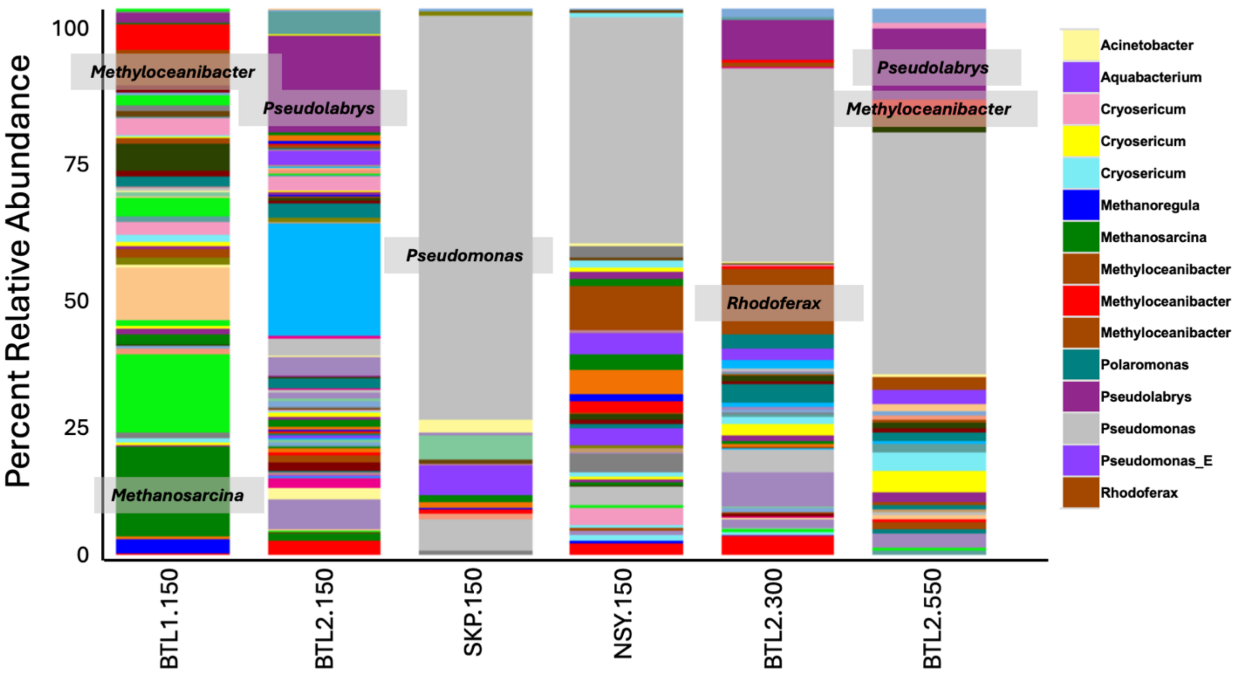

Supplemental Figure 4 Relative microbial composition at the genus level of core samples from metagenomic sequencing are featured in detail in Supplemental Table 3. Genera displayed in legend are are notable and all are >2% relative abundance in at least one site sample at specified depth.

Supplemental Table 3 Table of relative microbial composition at the genus level of core samples from metagenomic sequencing.

		Total	BTL1.150	BTL2.150	SKP 150	NSY.150	BTL2.300	BTL2.550
Legend	Taxonomy	%	%	%	%	%	%	%
	d__Archaea;p__Halobacteriota;o__Bog-38;f__Bog-38;g__Bog-38;s__Bog-38 sp003170935	1.4%	0.2%	2.6%	0.0%	2.1%	3.5%	0.0%
	d__Archaea;p__Halobacteriota;o__Methanomicrobiales;f__Methanoregulaceae;g__Methanoregula;s__Methanoregula sp003141335	0.5%	2.8%	0.0%	0.0%	0.4%	0.0%	0.0%
	d__Archaea;p__Halobacteriota;o__Methanomicrobiales;f__Methanosphaerulaceae;g__UBA288;s__UBA288 sp017883495	0.1%	0.4%	0.0%	0.0%	0.0%	0.0%	0.0%
	d__Archaea;p__Halobacteriota;o__Methanosarcinales;f__Methanosarcinaceae;g__Methanosarcina;s__Methanosarcina sp001714685	3.0%	16.4%	1.3%	0.0%	0.0%	0.0%	0.0%
	d__Archaea;p__Halobacteriota;o__Methanotrichales;f__Methanotrichaceae;g__Methanothrix;s__Methanothrix sp011391755	0.1%	0.2%	0.0%	0.0%	0.0%	0.1%	0.0%
	d__Archaea;p__Methanobacteriota;o__Methanobacteriales;f__Methanobacteriaceae;g__Methanobacterium_B;s__Methanobacterium_B sp000744455	0.1%	0.6%	0.0%	0.0%	0.0%	0.0%	0.0%
	d__Archaea;p__Thermoproteota;o__B26-1;f__UBA233;g__PALSA-986;s__PALSA-986 sp003141855	0.4%	1.0%	0.0%	0.0%	1.1%	0.6%	0.0%
	d__Archaea;p__Thermoproteota;o__B26-1;f__UBA233;g__PALSA-986;s__PALSA-986 sp003151735	0.0%	0.0%	0.1%	0.0%	0.0%	0.0%	0.0%
	d__Archaea;p__Thermoproteota;o__Nitrososphaerales;f__Nitrososphaeraceae;g__UBA10452;s__UBA10452 sp002501855	0.1%	0.0%	0.0%	0.0%	0.0%	0.0%	0.8%
	d__Bacteria;p__Acidobacteriota;o__Acidobacteriales;f__Koribacteraceae;g__Koribacter;s__Koribacter sp003151155	0.0%	0.0%	0.2%	0.0%	0.0%	0.0%	0.0%
	d__Bacteria;p__Acidobacteriota;o__Aminicenantales;f__RBG-16-66-30;g__RBG-16-66-30;s__RBG-16-66-30 sp001773855	0.1%	0.9%	0.0%	0.0%	0.0%	0.0%	0.0%
	d__Bacteria;p__Acidobacteriota;o__Aminicenantales;f__RBG-16-66-30;g__RBG-16-66-30;s__RBG-16-66-30 sp014894455	2.6%	14.4%	0.2%	0.0%	0.0%	0.5%	0.5%
	d__Bacteria;p__Acidobacteriota;o__Aminicenantales;f__RBG-16-66-30;g__RBG-16-66-30;s__RBG-16-66-30 sp017883305	0.2%	1.1%	0.0%	0.0%	0.0%	0.0%	0.0%
	d__Bacteria;p__Acidobacteriota;o__Gp7-AA8;f__Gp7-AA8;g__JADGNX01;s__JADGNX01 sp017883265	0.1%	0.4%	0.0%	0.0%	0.0%	0.0%	0.0%
	d__Bacteria;p__Acidobacteriota;o__Gp7-AA8;f__Gp7-AA8;g__QHVT01;s__QHVT01 sp017883215	0.0%	0.0%	0.2%	0.0%	0.0%	0.0%	0.0%
	d__Bacteria;p__Acidobacteriota;o__Pyrinomonadales;f__Pyrinomonadaceae;g__Pyrinomonas;s__Pyrinomonas methylaliphatogenes	0.0%	0.0%	0.0%	0.0%	0.0%	0.3%	0.0%
	d__Bacteria;p__Acidobacteriota;o__Vicinamibacterales;f__Fen-181;g__FEN-299;s__FEN-299 sp017883185	1.8%	0.0%	5.5%	0.0%	0.8%	1.5%	2.7%
	d__Bacteria;p__Acidobacteriota;o__Vicinamibacterales;f__UBA2999;g__JADGOC01;s__JADGOC01 sp017883165	0.4%	0.0%	2.0%	0.0%	0.0%	0.3%	0.0%
	d__Bacteria;p__Actinobacteriota;o__Acidimicrobiales;f__RAAP-2;g__Bog-756;s__Bog-756 sp003133325	0.0%	0.0%	0.1%	0.0%	0.0%	0.0%	0.0%
	d__Bacteria;p__Actinobacteriota;o__Acidimicrobiales;f__RAAP-2;g__Bog-756;s__Bog-756 sp003152015	0.4%	0.0%	1.8%	0.0%	0.0%	0.3%	0.0%
	d__Bacteria;p__Actinobacteriota;o__Acidimicrobiales;f__RAAP-2;g__Bog-756;s__Bog-756 sp017883065	0.0%	0.0%	0.1%	0.0%	0.0%	0.0%	0.0%
	d__Bacteria;p__Actinobacteriota;o__Acidimicrobiales;f__RAAP-2;g__Bog-756;s__Bog-756 sp017883075	0.0%	0.0%	0.1%	0.0%	0.0%	0.0%	0.0%
	d__Bacteria;p__Actinobacteriota;o__Acidimicrobiales;f__RAAP-2;g__CAIUMH01;s__CAIUMH01 sp017883125	0.0%	0.0%	0.1%	0.0%	0.0%	0.0%	0.0%
	d__Bacteria;p__Actinobacteriota;o__Acidimicrobiales;f__RAAP-2;g__RAAP-2;s__RAAP-2 sp017883045	0.0%	0.0%	0.2%	0.0%	0.0%	0.0%	0.0%
	d__Bacteria;p__Actinobacteriota;o__Actinomycetales;f__Cellulomonadaceae;g__Cellulomonas;s__Cellulomonas sp003115775	0.1%	0.0%	0.5%	0.0%	0.0%	0.0%	0.0%
	d__Bacteria;p__Actinobacteriota;o__Actinomycetales;f__Cellulomonadaceae;g__Cellulomonas;s__Cellulomonas sp012927595	0.2%	0.0%	0.3%	0.0%	0.0%	0.0%	0.8%

	d__Bacteria;p__Actinobacteriota;o__Actinomycetales;f__Cellulomonadaceae;g__JADGOR01;s__JADGOR01 sp017882855	0.3 %	0.0%	1.5%	0.0%	0.0%	0.5%	0.0%
	d__Bacteria;p__Actinobacteriota;o__Actinomycetales;f__Demequinaceae;g__JAFGBM01;s__JAFGBM01 sp017882825	0.1 %	0.2%	0.1%	0.0%	0.0%	0.0%	0.0%
	d__Bacteria;p__Actinobacteriota;o__Actinomycetales;f__Dermatophilaceae;g__UBA4719;s__UBA4719 sp003132125	0.5 %	0.0%	1.3%	0.0%	0.6%	0.3%	1.1%
	d__Bacteria;p__Actinobacteriota;o__Actinomycetales;f__Dermatophilaceae;g__UBA4719;s__UBA4719 sp012927555	0.2 %	0.0%	0.5%	0.0%	0.0%	0.0%	0.5%
	d__Bacteria;p__Actinobacteriota;o__Actinomycetales;f__Microbacteriaceae;g__Cryobacterium;s__Cryobacterium sp004402215	0.0 %	0.0%	0.2%	0.0%	0.0%	0.0%	0.0%
	d__Bacteria;p__Actinobacteriota;o__Actinomycetales;f__Micrococcaceae;g__Arthrobacter_I;s__Arthrobacter_I sp000427315	0.1 %	0.2%	0.6%	0.0%	0.0%	0.0%	0.0%
	d__Bacteria;p__Actinobacteriota;o__Actinomycetales;f__Micrococcaceae;g__Arthrobacter_I;s__Arthrobacter_I sp009928425	0.3 %	1.7%	0.4%	0.0%	0.0%	0.0%	0.0%
	d__Bacteria;p__Actinobacteriota;o__Actinomycetales;f__Micrococcaceae;g__Arthrobacter_I;s__Arthrobacter_I sp013359735	0.2 %	1.0%	0.0%	0.0%	0.0%	0.0%	0.0%
	d__Bacteria;p__Actinobacteriota;o__Actinomycetales;f__Micrococcaceae;g__Pseudarthrobacter;s__Pseudarthrobacter sulfonivorans	0.1 %	0.6%	0.0%	0.0%	0.0%	0.0%	0.0%
	d__Bacteria;p__Actinobacteriota;o__BMS3ABIN01;f__BMS3ABIN01;g__JACRMB01;s__JACRMB01 sp016650015	0.1 %	0.0%	0.0%	0.0%	0.4%	0.0%	0.0%
	d__Bacteria;p__Actinobacteriota;o__CADDZG01;f__WHSQ01;g__WHTI01;s__WHTI01 sp016649855	0.5 %	0.0%	0.0%	0.0%	3.0%	0.0%	0.0%
	d__Bacteria;p__Actinobacteriota;o__Gaiellales;f__Gaiellaceae;g__Fen-549;s__Fen-549 sp003132665	0.0 %	0.0%	0.2%	0.0%	0.0%	0.0%	0.0%
	d__Bacteria;p__Actinobacteriota;o__Gaiellales;f__Gaiellaceae;g__Fen-549;s__Fen-549 sp017882615	0.0 %	0.0%	0.1%	0.0%	0.0%	0.0%	0.0%
	d__Bacteria;p__Actinobacteriota;o__Gaiellales;f__Gaiellaceae;g__JACCTU01;s__JACCTU01 sp013812235	0.1 %	0.0%	0.0%	0.7%	0.0%	0.0%	0.0%
	d__Bacteria;p__Actinobacteriota;o__Humimicrobiales;f__Humimicrobiaceae;g__JAHILC01;s__JAHILC01 sp018897225	0.3 %	1.1%	0.0%	0.0%	0.6%	0.0%	0.0%
	d__Bacteria;p__Actinobacteriota;o__Miltoncostaeales;f__Miltoncostaeaceae;g__Ga0077560;s__Ga0077560 sp017882555	0.0 %	0.0%	0.1%	0.0%	0.0%	0.0%	0.0%
	d__Bacteria;p__Actinobacteriota;o__Mycobacteriales;f__JADGOU01;g__JADGOU01;s__JADGOU01 sp017882835	0.2 %	0.0%	0.4%	0.0%	0.0%	0.8%	0.0%
	d__Bacteria;p__Actinobacteriota;o__Mycobacteriales;f__JADGOV01;g__JADGOV01;s__JADGOV01 sp017882785	1.7 %	9.7%	0.0%	0.0%	0.0%	0.0%	0.8%
	d__Bacteria;p__Actinobacteriota;o__Mycobacteriales;f__Mycobacteriaceae;g__X156;s__X156 sp017882745	0.1 %	0.0%	0.5%	0.0%	0.0%	0.2%	0.0%
	d__Bacteria;p__Actinobacteriota;o__OPB41;f__PALSA-660;g__PALSA-660;s__PALSA-660 sp003133285	1.1 %	0.0%	0.0%	0.0%	0.0%	6.3%	0.0%
	d__Bacteria;p__Actinobacteriota;o__Propionibacteriales;f__Nocardioidaceae;g__JABFXA01;s__JABFXA01 sp019039255	0.1 %	0.3%	0.0%	0.0%	0.0%	0.0%	0.0%
	d__Bacteria;p__Actinobacteriota;o__Propionibacteriales;f__Propionibacteriaceae;g__Cutibacterium;s__Cutibacterium acnes	2.4 %	0.0%	0.0%	6.0%	3.4%	4.3%	0.7%
	d__Bacteria;p__Actinobacteriota;o__RBG-16-64-13;f__RBG-16-64-13;g__Chersky-299;s__Chersky-299 sp017882525	0.0 %	0.2%	0.0%	0.0%	0.0%	0.0%	0.0%
	d__Bacteria;p__Actinobacteriota;o__Solirubrobacterales;f__70-9;g__VAYN01;s__VAYN01 sp017882505	0.1 %	0.0%	0.4%	0.0%	0.0%	0.3%	0.0%
	d__Bacteria;p__Actinobacteriota;o__Solirubrobacterales;f__Solirubrobacteraceae;g__Palsa-465;s__Palsa-465 sp017882405	0.1 %	0.0%	0.0%	0.0%	0.0%	0.0%	0.5%
	d__Bacteria;p__Actinobacteriota;o__Solirubrobacterales;f__Solirubrobacteraceae;g__Palsa-465;s__Palsa-465 sp017882465	0.2 %	1.3%	0.0%	0.0%	0.0%	0.0%	0.0%
	d__Bacteria;p__Actinobacteriota;o__Solirubrobacterales;f__Solirubrobacteraceae;g__Palsa-744;s__Palsa-744 sp017882375	0.1 %	0.0%	0.4%	0.0%	0.0%	0.0%	0.0%
	d__Bacteria;p__Actinobacteriota;o__Solirubrobacterales;f__Solirubrobacteraceae;g__Palsa-744;s__Palsa-744 sp017882445	0.0 %	0.0%	0.1%	0.0%	0.0%	0.0%	0.0%
	d__Bacteria;p__Actinobacteriota;o__UBA2241;f__UBA2241;g__CAIXSE01;s__CAIXSE01 sp017882325	0.1 %	0.0%	0.1%	0.0%	0.0%	0.0%	0.8%
	d__Bacteria;p__Actinobacteriota;o__UBA2241;f__UBA2241;g__PALSA-647;s__PALSA-647 sp003132585	0.0 %	0.0%	0.2%	0.0%	0.0%	0.0%	0.0%
	d__Bacteria;p__Atribacterota;o__SB-45;f__34-128;g__34-128;s__34-128 sp014894735	0.1 %	0.0%	0.0%	0.0%	0.4%	0.0%	0.0%

	d__Bacteria;p__Atribacterota;o__SB-45;f__34-128;g__CG2-30-33-13;s__CG2-30-33-13 sp002782675	0.4 %	1.6%	0.2%	0.0%	0.0%	0.2%	0.5%
	d__Bacteria;p__Bacteroidota;o__Bacteroidales;f__FEN-979;g__FEN-979;s__FEN-979 sp017882335	0.0 %	0.0%	0.2%	0.0%	0.0%	0.0%	0.0%
	d__Bacteria;p__Bacteroidota;o__Bacteroidales;f__Paludibacteraceae;g__Paludibacter;s__Paludibacter sp017882285	0.0 %	0.0%	0.2%	0.0%	0.0%	0.0%	0.0%
	d__Bacteria;p__Bacteroidota;o__Bacteroidales;f__VadinHA17;g__LD21;s__LD21 sp017882345	0.2 %	0.0%	0.6%	0.0%	0.0%	0.6%	0.0%
	d__Bacteria;p__Bacteroidota;o__Chitinophagales;f__Chitinophagaceae;g__Ginsengibacter;s__Ginsengibacter sp017882215	0.4 %	0.0%	1.3%	0.0%	0.5%	0.5%	0.0%
	d__Bacteria;p__Caldisericota;o__Cryosericales;f__Cryosericaceae;g__Cryosericum;s__Cryosericum hinesii	0.7 %	0.4%	0.4%	0.0%	0.5%	1.0%	1.7%
	d__Bacteria;p__Caldisericota;o__Cryosericales;f__Cryosericaceae;g__Cryosericum;s__Cryosericum odellii	1.4 %	0.8%	0.8%	0.0%	0.3%	2.2%	4.1%
	d__Bacteria;p__Caldisericota;o__Cryosericales;f__Cryosericaceae;g__Cryosericum;s__Cryosericum septentrionale	1.2 %	1.2%	0.4%	0.0%	0.8%	1.4%	3.3%
	d__Bacteria;p__Caldisericota;o__Cryosericales;f__Cryosericaceae;g__Cryosericum;s__Cryosericum sp013824685	0.4 %	2.4%	0.1%	0.0%	0.0%	0.0%	0.0%
	d__Bacteria;p__Caldisericota;o__Cryosericales;f__Cryosericaceae;g__Cryosericum;s__Cryosericum terrychapinii	0.5 %	1.0%	0.2%	0.0%	0.0%	0.5%	1.6%
	d__Bacteria;p__Campylobacterota;o__Campylobacterales;f__Sulfurospirillaceae;g__Sulfurospirillum;s__Sulfurospirillum arsenophilum	0.0 %	0.0%	0.1%	0.0%	0.0%	0.0%	0.0%
	d__Bacteria;p__Chloroflexota;o__Anaerolineales;f__EnvOPS12;g__UBA12087;s__UBA12087 sp001796335	0.1 %	0.0%	0.0%	0.9%	0.0%	0.0%	0.0%
	d__Bacteria;p__Chloroflexota;o__Anaerolineales;f__EnvOPS12;g__UBA877;s__UBA877 sp017882065	0.6 %	0.0%	0.2%	0.0%	3.4%	0.3%	0.0%
	d__Bacteria;p__Chloroflexota;o__Anaerolineales;f__EnvOPS12;g__UBA877;s__UBA877 sp017882125	0.6 %	3.5%	0.1%	0.0%	0.0%	0.0%	0.0%
	d__Bacteria;p__Chloroflexota;o__CG2-30-64-16;f__CG2-30-64-16;g__JADGQF01;s__JADGQF01 sp017882045	0.2 %	0.0%	1.0%	0.0%	0.0%	0.4%	0.0%
	d__Bacteria;p__Chloroflexota;o__Dehalococcoidales;f__RBG-16-60-22;g__Fen-1077;s__Fen-1077 sp003141235	0.0 %	0.2%	0.0%	0.0%	0.0%	0.0%	0.0%
	d__Bacteria;p__Chloroflexota;o__Limnocylindrales;f__CSP1-4;g__CTSoil-043;s__CTSoil-043 sp017881985	0.3 %	0.9%	0.7%	0.0%	0.0%	0.2%	0.0%
	d__Bacteria;p__Chloroflexota;o__Limnocylindrales;f__CSP1-4;g__CTSoil-043;s__CTSoil-043 sp017882025	0.3 %	0.0%	1.0%	0.0%	0.4%	0.5%	0.0%
	d__Bacteria;p__Chloroflexota;o__Limnocylindrales;f__CSP1-4;g__Fen-1039;s__Fen-1039 sp003141245	0.1 %	0.3%	0.0%	0.0%	0.0%	0.0%	0.0%
	d__Bacteria;p__Chloroflexota;o__Limnocylindrales;f__CSP1-4;g__Fen-1039;s__Fen-1039 sp003141455	0.1 %	0.3%	0.4%	0.0%	0.0%	0.0%	0.0%
	d__Bacteria;p__Chloroflexota;o__Limnocylindrales;f__CSP1-4;g__Palsa-1033;s__Palsa-1033 sp017881915	0.0 %	0.0%	0.3%	0.0%	0.0%	0.0%	0.0%
	d__Bacteria;p__Deinococcota;o__Deinococcales;f__Thermaceae;g__Meiothermus_B;s__Meiothermus_B silvanus	0.2 %	0.0%	0.0%	0.0%	0.0%	0.7%	0.4%
	d__Bacteria;p__Desulfobacterota;o__Desulfobaccales;f__0-14-0-80-60-11;g__0-14-0-80-60-11;s__0-14-0-80-60-11 sp002779455	0.1 %	0.0%	0.0%	0.0%	0.7%	0.0%	0.0%
	d__Bacteria;p__Desulfobacterota;o__Desulfobaccales;f__0-14-0-80-60-11;g__0-14-0-80-60-11;s__0-14-0-80-60-11 sp016875015	0.1 %	0.0%	0.0%	0.0%	0.5%	0.0%	0.0%
	d__Bacteria;p__Desulfobacterota;o__Desulfobaccales;f__0-14-0-80-60-11;g__0-14-0-80-60-11;s__0-14-0-80-60-11 sp018897875	0.5 %	0.0%	0.0%	0.0%	3.2%	0.0%	0.0%
	d__Bacteria;p__Desulfobacterota;o__Syntrophales;f__Fen-1087;g__Fen-1087;s__Fen-1087 sp003161855	0.0 %	0.2%	0.0%	0.0%	0.0%	0.0%	0.0%
	d__Bacteria;p__Desulfobacterota;o__Syntrophales;f__Smithellaceae;g__FEN-1160;s__FEN-1160 sp003142835	1.6 %	1.9%	1.9%	0.0%	0.8%	3.4%	1.7%
	d__Bacteria;p__Desulfobacterota;o__Syntrophales;f__UBA5619;g__UBA5619;s__UBA5619 sp001873745	0.5 %	1.1%	0.0%	0.0%	0.7%	0.6%	0.7%
	d__Bacteria;p__Desulfobacterota;o__Syntrophales;f__UBA5619;g__UBA5619;s__UBA5619 sp003141195	1.4 %	4.9%	0.3%	0.0%	1.0%	1.0%	1.1%
	d__Bacteria;p__Desulfobacterota;o__Syntrophales;f__UBA5619;g__UBA5619;s__UBA5619 sp018896355	0.4 %	1.0%	0.0%	0.0%	0.4%	0.4%	0.4%
	d__Bacteria;p__Desulfobacterota_E;o__MBNT15;f__MBNT15;g__CG2-30-66-27;s__CG2-30-66-27 sp001873935	0.4 %	0.0%	0.0%	0.7%	2.0%	0.0%	0.0%

	d__Bacteria;p__Desulfobacterota_E;o__MBNT15;f__MBNT15;g__CG2-30-66-27;s__CG2-30-66-27 sp002279275	0.3 %	0.0%	0.0%	0.4%	1.4%	0.0%	0.0%
	d__Bacteria;p__Desulfobacterota_E;o__MBNT15;f__MBNT15;g__CG2-30-66-27;s__CG2-30-66-27 sp011391555	0.9 %	0.0%	0.0%	1.0%	4.5%	0.0%	0.0%
	d__Bacteria;p__Desulfobacterota_E;o__MBNT15;f__MBNT15;g__CG2-30-66-27;s__CG2-30-66-27 sp017881405	0.7 %	0.0%	0.0%	1.3%	2.8%	0.0%	0.0%
	d__Bacteria;p__Dormibacterota;o__UBA8260;f__UBA8260;g__Palsa-875;s__Palsa-875 sp003153125	0.0 %	0.0%	0.2%	0.0%	0.0%	0.0%	0.0%
	d__Bacteria;p__Firmicutes;o__Alicyclobacillales;f__Alicyclobacillaceae;g__Alicyclobacillus_A;s__Alicyclobacillus_A pomorum	0.0 %	0.2%	0.0%	0.0%	0.0%	0.0%	0.0%
	d__Bacteria;p__Firmicutes;o__Bacillales_B;f__DSM-1321;g__Peribacillus;s__Peribacillus psychrosaccharolyticus	0.0 %	0.3%	0.0%	0.0%	0.0%	0.0%	0.0%
	d__Bacteria;p__Firmicutes;o__Bacillales_B;f__Domibacillaceae;g__Domibacillus;s__Domibacillus tundrae	0.5 %	3.2%	0.0%	0.0%	0.0%	0.0%	0.0%
	d__Bacteria;p__Firmicutes;o__ML615J-28;f__CAG-698;g__UBA2253;s__UBA2253 sp002347755	0.1 %	0.2%	0.0%	0.0%	0.0%	0.2%	0.0%
	d__Bacteria;p__Firmicutes;o__Paenibacillales;f__Paenibacillaceae;g__Paenibacillus_C;s__Paenibacillus_C sp001956295	0.2 %	1.1%	0.0%	0.0%	0.0%	0.0%	0.0%
	d__Bacteria;p__Firmicutes_A;o__Clostridiales;f__Clostridiaceae;g__Clostridium_AD;s__Clostridium_AD psychrophilum	0.2 %	1.0%	0.0%	0.0%	0.0%	0.0%	0.0%
	d__Bacteria;p__Firmicutes_A;o__Clostridiales;f__Clostridiaceae;g__Clostridium_AD;s__Clostridium_AD sp002403785	0.3 %	2.0%	0.0%	0.0%	0.0%	0.0%	0.0%
	d__Bacteria;p__Firmicutes_B;o__Desulfitobacteriales;f__Desulfitobacteriaceae;g__Desulfosporosinus;s__Desulfosporosinus fructosivorans	0.1 %	0.0%	0.0%	0.0%	0.0%	0.0%	0.8%
	d__Bacteria;p__Firmicutes_B;o__Desulfitobacteriales;f__Desulfitobacteriaceae;g__Desulfosporosinus;s__Desulfosporosinus sp002413075	0.1 %	0.0%	0.0%	0.0%	0.0%	0.0%	0.8%
	d__Bacteria;p__Firmicutes_B;o__Desulfitobacteriales;f__Desulfitobacteriaceae;g__Desulfosporosinus;s__Desulfosporosinus sp003132105	0.2 %	0.0%	0.0%	0.0%	0.0%	0.0%	1.3%
	d__Bacteria;p__Gemmatimonadota;o__Gemmatimonadales;f__GWC2-71-9;g__Palsa-1233;s__Palsa-1233 sp003169895	0.0 %	0.0%	0.1%	0.0%	0.0%	0.0%	0.0%
	d__Bacteria;p__Gemmatimonadota;o__Gemmatimonadales;f__Gemmatimonadaceae;g__FEN-1250;s__FEN-1250 sp003142495	0.6 %	0.0%	3.3%	0.0%	0.0%	0.2%	0.0%
	d__Bacteria;p__Gemmatimonadota;o__Gemmatimonadales;f__Gemmatimonadaceae;g__Fen-1231;s__Fen-1231 sp003171215	0.0 %	0.0%	0.3%	0.0%	0.0%	0.0%	0.0%
	d__Bacteria;p__Gemmatimonadota;o__Gemmatimonadales;f__Gemmatimonadaceae;g__Fen-1231;s__Fen-1231 sp017881585	0.6 %	0.0%	3.2%	0.0%	0.0%	0.5%	0.0%
	d__Bacteria;p__Gemmatimonadota;o__Gemmatimonadales;f__Gemmatimonadaceae;g__UBA4720;s__UBA4720 sp003131365	0.1 %	0.0%	0.5%	0.0%	0.0%	0.0%	0.0%
	d__Bacteria;p__Gemmatimonadota;o__Gemmatimonadales;f__Gemmatimonadaceae;g__UBA4720;s__UBA4720 sp017881465	3.7 %	0.0%	20.6 %	0.0%	0.0%	1.6%	0.0%
	d__Bacteria;p__Gemmatimonadota;o__Gemmatimonadales;f__Gemmatimonadaceae;g__UBA4720;s__UBA4720 sp017881685	0.0 %	0.0%	0.2%	0.0%	0.0%	0.0%	0.0%
	d__Bacteria;p__Proteobacteria;o__Acetobacterales;f__Acetobacteraceae;g__PALSA-911;s__PALSA-911 sp003133265	0.1 %	0.0%	0.9%	0.0%	0.0%	0.0%	0.0%
	d__Bacteria;p__Proteobacteria;o__Burkholderiales;f__Burkholderiaceae;g__Aquabacterium;s__Aquabacterium parvum	2.3 %	0.0%	0.0%	5.5%	3.8%	2.3%	2.5%
	d__Bacteria;p__Proteobacteria;o__Burkholderiales;f__Burkholderiaceae;g__Melaminivora;s__Melaminivora alkalimesophila	0.1 %	0.0%	0.0%	0.3%	0.6%	0.0%	0.0%
	d__Bacteria;p__Proteobacteria;o__Burkholderiales;f__Burkholderiaceae;g__Polaromonas;s__Polaromonas sp000688115	0.9 %	0.0%	2.6%	0.0%	0.0%	2.6%	0.0%
	d__Bacteria;p__Proteobacteria;o__Burkholderiales;f__Burkholderiaceae;g__Polaromonas;s__Polaromonas sp002381615	0.1 %	0.0%	0.3%	0.0%	0.0%	0.0%	0.0%
	d__Bacteria;p__Proteobacteria;o__Burkholderiales;f__Burkholderiaceae;g__Ramlibacter;s__Ramlibacter tataouinensis_A	0.1 %	0.0%	0.6%	0.0%	0.0%	0.0%	0.0%
	d__Bacteria;p__Proteobacteria;o__Burkholderiales;f__Burkholderiaceae;g__Rhodoferax;s__Rhodoferax ferrireducens	3.8 %	0.0%	0.2%	0.0%	8.0%	12.0 %	2.5%
	d__Bacteria;p__Proteobacteria;o__Burkholderiales;f__Burkholderiaceae;g__Rhodoferax;s__Rhodoferax sp017880985	0.1 %	0.0%	0.1%	0.0%	0.0%	0.5%	0.0%
	d__Bacteria;p__Proteobacteria;o__Burkholderiales;f__Casimicrobiaceae;g__PALSA-1003;s__PALSA-1003 sp017880805	0.0 %	0.0%	0.2%	0.0%	0.0%	0.0%	0.0%
	d__Bacteria;p__Proteobacteria;o__Burkholderiales;f__Casimicrobiaceae;g__PALSA-1005;s__PALSA-1005 sp003153335	0.0 %	0.0%	0.2%	0.0%	0.0%	0.0%	0.0%

	d__Bacteria;p__Proteobacteria;o__Burkholderiales;f__Gallionellaceae;g__Gallionella; s__Gallionella sp001801055	0.2%	0.0%	0.0%	0.0%	1.4%	0.0%	0.0%
	d__Bacteria;p__Proteobacteria;o__Burkholderiales;f__Gallionellaceae;g__Gallionella; s__Gallionella sp003134595	0.3%	0.0%	0.3%	0.0%	1.3%	0.0%	0.0%
	d__Bacteria;p__Proteobacteria;o__Burkholderiales;f__Gallionellaceae;g__Gallionella; s__Gallionella sp003153155	0.2%	0.0%	0.4%	0.0%	0.8%	0.0%	0.0%
	d__Bacteria;p__Proteobacteria;o__Burkholderiales;f__Gallionellaceae;g__Gallionella; s__Gallionella sp016183575	0.2%	0.0%	0.0%	0.0%	1.2%	0.0%	0.0%
	d__Bacteria;p__Proteobacteria;o__Burkholderiales;f__Gallionellaceae;g__Gallionella; s__Gallionella sp017880945	0.4%	0.0%	2.6%	0.0%	0.0%	0.1%	0.0%
	d__Bacteria;p__Proteobacteria;o__Burkholderiales;f__Methylophilaceae;g__JADGRN01;s__JADGRN01 sp017880825	0.1%	0.0%	0.2%	0.0%	0.0%	0.1%	0.0%
	d__Bacteria;p__Proteobacteria;o__Burkholderiales;f__Rhodocyclaceae;g__Hydrogenophilus;s__Hydrogenophilus thermoluteolus	0.2%	0.0%	0.0%	0.6%	0.6%	0.1%	0.0%
	d__Bacteria;p__Proteobacteria;o__Burkholderiales;f__SG8-39;g__2-12-FULL-64-23;s__2-12-FULL-64-23 sp017880745	0.4%	0.0%	0.2%	0.0%	2.0%	0.0%	0.0%
	d__Bacteria;p__Proteobacteria;o__Burkholderiales;f__SG8-39;g__JADGRQ01;s__JADGRQ01 sp017880775	0.0%	0.0%	0.2%	0.0%	0.0%	0.0%	0.0%
	d__Bacteria;p__Proteobacteria;o__Burkholderiales;f__SG8-41;g__PALSA-1004;s__PALSA-1004 sp003153455	0.1%	0.0%	0.8%	0.0%	0.0%	0.0%	0.0%
	d__Bacteria;p__Proteobacteria;o__Burkholderiales;f__Usitatibacteraceae;g__Usitatibacter;s__Usitatibacter sp017880705	0.1%	0.4%	0.0%	0.0%	0.0%	0.0%	0.0%
	d__Bacteria;p__Proteobacteria;o__Caulobacterales;f__Caulobacteraceae;g__Phenylobacterium;s__Phenylobacterium sp003136395	0.1%	0.0%	0.4%	0.0%	0.0%	0.0%	0.0%
	d__Bacteria;p__Proteobacteria;o__Enterobacterales;f__Enterobacteriaceae;g__Pantoea;s__Pantoea dispersa	0.7%	0.0%	0.0%	4.4%	0.0%	0.0%	0.0%
	d__Bacteria;p__Proteobacteria;o__Enterobacterales;f__Enterobacteriaceae;g__Siccibacter;s__Siccibacter colletis	0.1%	0.0%	0.0%	0.6%	0.0%	0.0%	0.0%
	d__Bacteria;p__Proteobacteria;o__Pseudomonadales;f__Moraxellaceae;g__Acinetobacter;s__Acinetobacter johnsonii	0.6%	0.0%	0.0%	2.3%	0.7%	0.4%	0.4%
	d__Bacteria;p__Proteobacteria;o__Pseudomonadales;f__Pseudomonadaceae;g__Pseudomonas;s__Pseudomonas aeruginosa	32.5%	0.0%	0.0%	74.0%	41.3%	35.3%	44.2%
	d__Bacteria;p__Proteobacteria;o__Pseudomonadales;f__Pseudomonadaceae;g__Pseudomonas_A;s__Pseudomonas_A stutzeri	0.0%	0.0%	0.0%	0.0%	0.0%	0.3%	0.0%
	d__Bacteria;p__Proteobacteria;o__Pseudomonadales;f__Pseudomonadaceae;g__Pseudomonas_E;s__Pseudomonas_E mandelii	0.0%	0.0%	0.1%	0.0%	0.0%	0.0%	0.0%
	d__Bacteria;p__Proteobacteria;o__Pseudomonadales;f__Pseudomonadaceae;g__Pseudomonas_E;s__Pseudomonas_E mandelii_C	0.0%	0.0%	0.2%	0.0%	0.0%	0.0%	0.0%
	d__Bacteria;p__Proteobacteria;o__Pseudomonadales;f__Pseudomonadaceae;g__Pseudomonas_E;s__Pseudomonas_E monteilii_A	0.1%	0.0%	0.0%	0.8%	0.0%	0.0%	0.0%
	d__Bacteria;p__Proteobacteria;o__Pseudomonadales;f__Pseudomonadaceae;g__Pseudomonas_E;s__Pseudomonas_E sp001297125	0.4%	0.0%	2.6%	0.0%	0.0%	0.0%	0.0%
	d__Bacteria;p__Proteobacteria;o__Pseudomonadales;f__Pseudomonadaceae;g__Pseudomonas_E;s__Pseudomonas_E sp003151075	0.1%	0.0%	0.4%	0.0%	0.0%	0.0%	0.0%
	d__Bacteria;p__Proteobacteria;o__Pseudomonadales;f__Pseudomonadaceae;g__Pseudomonas_E;s__Pseudomonas_E sp012935755	0.0%	0.0%	0.1%	0.0%	0.0%	0.0%	0.0%
	d__Bacteria;p__Proteobacteria;o__Rhizobiales;f__Hyphomicrobiaceae;g__Hyphomicrobium;s__Hyphomicrobium facile	0.2%	1.3%	0.0%	0.0%	0.0%	0.0%	0.0%
	d__Bacteria;p__Proteobacteria;o__Rhizobiales;f__Methyloligellaceae;g__Methyloceanibacter;s__Methyloceanibacter sp002383105	0.2%	0.0%	0.0%	0.0%	0.0%	0.0%	1.2%
	d__Bacteria;p__Proteobacteria;o__Rhizobiales;f__Methyloligellaceae;g__Methyloceanibacter;s__Methyloceanibacter sp003135455	1.7%	6.7%	0.6%	0.0%	0.0%	0.7%	2.0%
	d__Bacteria;p__Proteobacteria;o__Rhizobiales;f__Methyloligellaceae;g__Methyloceanibacter;s__Methyloceanibacter sp016649675	1.4%	4.5%	0.3%	0.0%	0.0%	0.6%	2.8%
	d__Bacteria;p__Proteobacteria;o__Rhizobiales;f__Xanthobacteraceae;g__Bradyrhizobium;s__Bradyrhizobium erythrophlei_D	0.1%	0.0%	0.6%	0.0%	0.0%	0.0%	0.0%
	d__Bacteria;p__Proteobacteria;o__Rhizobiales;f__Xanthobacteraceae;g__Bradyrhizobium;s__Bradyrhizobium sp004799445	0.1%	0.0%	0.8%	0.0%	0.0%	0.0%	0.0%
	d__Bacteria;p__Proteobacteria;o__Rhizobiales;f__Xanthobacteraceae;g__Bradyrhizobium;s__Bradyrhizobium sp017881085	0.2%	0.4%	0.6%	0.0%	0.0%	0.0%	0.0%
	d__Bacteria;p__Proteobacteria;o__Rhizobiales;f__Xanthobacteraceae;g__Pseudolabrys;s__Pseudolabrys sp003133345	6.6%	1.9%	17.6%	0.0%	0.0%	7.2%	13.0%

	d__Bacteria;p__Proteobacteria;o__Rhizobiales;f__Xanthobacteraceae;g__Pseudolabrys;s__Pseudolabrys sp003152455	0.0 %	0.0%	0.2%	0.0%	0.0%	0.0%	0.0%
	d__Bacteria;p__Proteobacteria;o__Rhizobiales;f__Xanthobacteraceae;g__Pseudolabrys;s__Pseudolabrys sp016185205	0.2 %	0.0%	0.2%	0.0%	0.7%	0.0%	0.0%
	d__Bacteria;p__Proteobacteria;o__Rhizobiales;f__Xanthobacteraceae;g__Z2-YC6860;s__Z2-YC6860 sp003169835	0.2 %	0.0%	0.0%	0.0%	0.0%	0.0%	1.1%
	d__Bacteria;p__Proteobacteria;o__Xanthomonadales;f__Rhodanobacteraceae;g__Dokdonella_A;s__Dokdonella_A sp003142815	0.8 %	0.0%	4.0%	0.0%	0.0%	0.5%	0.0%
	d__Bacteria;p__Proteobacteria;o__Xanthomonadales;f__Xanthomonadaceae;g__Pseudoxanthomonas;s__Pseudoxanthomonas taiwanensis	0.1 %	0.0%	0.0%	0.0%	0.4%	0.0%	0.0%
	d__Bacteria;p__Ratteibacteria;o__MWAK01;f__MWAK01;g__JAHJUB01;s__JAHJUB01 sp018829455	0.0 %	0.1%	0.0%	0.0%	0.0%	0.0%	0.0%
	d__Bacteria;p__Verrucomicrobiota;o__Chthoniobacterales;f__UBA10450;g__AV80;s__AV80 sp003167555	0.1 %	0.5%	0.2%	0.0%	0.0%	0.0%	0.0%
	d__Bacteria;p__Verrucomicrobiota;o__JAAZAB01;f__VSJD01;g__VSJD01;s__VSJD01 sp018896395	0.0 %	0.0%	0.0%	0.0%	0.0%	0.2%	0.0%
	d__Bacteria;p__Verrucomicrobiota;o__Pedosphaerales;f__JADGRT01;g__JADGRT01;s__JADGRT01 sp017880715	0.9 %	0.0%	0.2%	0.5%	0.4%	1.5%	2.6%
	d__Bacteria;p__Verrucomicrobiota;o__Pedosphaerales;f__UBA11358;g__UBA11358;s__UBA11358 sp003455565	0.0 %	0.0%	0.2%	0.0%	0.0%	0.0%	0.0%

Samples	Number of raw reads	Reads surviving (%)	Number of generated contigs
BTL1.150	24,895,989	94.42	1,284,200
BTL2.150	24,248,877	92.26	1,268,986
SKP.150	21,124,080	96.93	1,501,248
NSY.150	27,289,809	84.51	1,159,402
BTL2.300	24,931,253	77.93	588,060
BTL2.550	28,421,605	87.29	1,082,594

Supplemental Table 4 Metagenomic read processing summary for each core at various depths. Adjustments to naming occurred since this table was generated and cores site alternative naming is as follows: BTL1.500=BTL.1.3, BTL1.150=BTL.2.3, SKP.150=SL.1.3, NSY.150=NSY.1.3, BTL2.300=BTL.2.6, BTL2.550=BTL.2.11

Table Analyzed	Row statistics of Grouped: Multiple t tests				
Data sets analyzed	A-E				
**ANOVA summary**					
F	2.523				
P value	0.0422				
P value summary	*				
Significant diff. among means (P < 0.05)?	Yes				
R squared	0.04758				
**Brown-Forsythe test**					
F (DFn, DFd)	2.413 (4, 202)				
P value	0.0503				
P value summary	ns				
Are SDs significantly different (P < 0.05)?	No				
**Bartlett's test**					
Bartlett's statistic (corrected)	317.1				
P value	<0.0001				
P value summary	****				
Are SDs significantly different (P < 0.05)?	Yes				
**ANOVA table**	**SS**	**DF**	**MS**	**F (DFn, DFd)**	**P value**
Treatment (between columns)	3.219e+016	4	8.047e+015	F (4, 202) = 2.	P=0.0422
Residual (within columns)	6.443e+017	202	3.189e+015		
Total	6.765e+017	206			
**Data summary**					
Number of treatments (columns)	5				
Number of values (total)	207				

Supplemental Table 5 One-way analysis of variance (ANOVA) or Kruskal-Wallis test was performed on averaged VOC samples from each borehole. The Brown-Forsythe and Bartlett's test were also performed as part of the ANOVA analysis.

Pathway	KEGG Enzyme	Reaction	BTL1.150	BTL2.150	SKP.150	NSY.150	BTL2.300	BTL2.550
Propanoate metabolism	acetate---CoA ligase (ADP-forming) subunit alpha [EC:6.2.1.13]	R00920	332	127	183	144	142	113
	acetate kinase [EC:2.7.2.1]<br>propionate kinase [EC:2.7.2.15]	R01353	152	185	209	186	82	191
	phosphate propanoyltransferase [EC:2.3.1.222]	R00921	116	129	100	63	53	104
	2-oxoisovalerate dehydrogenase E2 component (dihydrolipoyl transacylase) [EC:2.3.1.168]	R10998	17	40	75	42	15	17
	dihydrolipoyl dehydrogenase [EC:1.8.1.4]	R08549	638	860	993	770	278	555
	pyruvate ferredoxin oxidoreductase alpha subunit [EC:1.2.7.1]	R08034	711	487	431	497	408	389
	2-oxoisovalerate/pyruvate ferredoxin oxidoreductase gamma subunit [EC:1.2.7.7 1.2.7.1]	R08567	125	26	36	52	25	29
	formate C-acetyltransferase [EC:2.3.1.54]	R06987	263	102	80	64	122	257
	butyryl-CoA dehydrogenase [EC:1.3.8.1]	R04751	450	892	668	556	198	291
	acrylyl-CoA reductase (NADPH) [EC:1.3.1.-]	R00919	21	42	14	20	11	11
	lactoyl-CoA dehydratase subunit alpha [EC:4.2.1.54]	R02963	4	2	3	2	2	7
	3-hydroxyacyl-CoA dehydrogenase / enoyl-CoA hydratase / 3-hydroxybutyryl-CoA epimerase / enoyl-CoA isomerase [EC:1.1.1.35 4.2.1.17 5.1.2.3 5.3.3.8]	R08094	111	183	138	99	26	57
	multifunctional beta-oxidation protein [EC:4.2.1.- 1.1.1.-]	R03045	222	456	333	254	87	128
	3-hydroxyisobutyryl-CoA hydrolase [EC:3.1.2.4]	R05064	13	19	2	4	1	5
	3-hydroxyisobutyrate/3-hydroxypropionate dehydrogenase [EC:1.1.1.31 1.1.1.59]	R05066	299	485	304	245	91	186
	acetyl-CoA/propionyl-CoA carboxylase carboxyl transferase subunit [EC:6.4.1.2 6.4.1.3 2.1.3.15]	R04386	211	215	284	211	82	145
	malonyl-CoA decarboxylase [EC:4.1.1.9]	R00233	24	30	11	27	7	8
	methylmalonyl-CoA carboxyltransferase 5S subunit [EC:2.1.3.1]	R00930	22	8	4	2	9	9
	4-aminobutyrate aminotransferase / (S)-3-amino-2-methylpropionate transaminase / 5-aminovalerate transaminase [EC:2.6.1.19 2.6.1.22 2.6.1.48]	R04188	128	125	210	73	46	80
	beta-alanine---2-oxoglutarate transaminase [EC:2.6.1.120]	R00908	220	228	449	172	84	128
	propionyl-CoA carboxylase alpha chain [EC:6.4.1.3], propionyl-CoA carboxylase beta chain [EC:6.4.1.3 2.1.3.15]	R01859	279	380	473	277	94	220
	ethylmalonyl-CoA/methylmalonyl-CoA decarboxylase [EC:4.1.1.94 4.1.1.-]	R00923	14	22	3	18	5	5
	methylmalonyl-CoA/ethylmalonyl-CoA epimerase [EC:5.1.99.1]	R09979	82	64	117	40	29	55
	succinyl-CoA synthetase alpha subunit [EC:6.2.1.4 6.2.1.5]	R00727	1	0	0	0	1	1
	malonate-semialdehyde dehydrogenase (acetylating) / methylmalonate-semialdehyde dehydrogenase [EC:1.2.1.18 1.2.1.27]	R00935	120	160	207	117	44	87
	2-methylcitrate synthase [EC:2.3.3.5]	R00931	28	37	25	37	14	40
	2-methylcitrate dehydratase (2-methyl-trans-aconitate forming) [EC:4.2.1.117]	R11263	1	2	8	11	8	11
	aconitate hydratase 2 / 2-methylisocitrate dehydratase [EC:4.2.1.3 4.2.1.99]	R04425	18	19	26	61	18	9
	methylisocitrate lyase [EC:4.1.3.30]	R00409	14	36	22	35	8	10
	methylglyoxal synthase [EC:4.2.3.3]	R01016	20	27	11	12	8	20
	methylglyoxal reductase [EC:1.1.1.-]	R10718	4	9	12	6	6	9
	glycerol dehydrogenase [EC:1.1.1.6]	R10717	5	6	2	1	5	8
	1,3-propanediol dehydrogenase [EC:1.1.1.202]	R03119	21	2	8	0	7	10
	propionaldehyde dehydrogenase [EC:1.2.1.87]	R09097	31	19	30	13	16	15
Styrene degradation	styrene monooxygenase [EC:1.14.14.11]; styrene monooxygenase reductase component [EC:1.5.1.-]	R05488	0	0	2	0	5	1
	styrene-oxide isomerase [EC:5.3.99.7]	R10697	0	0	2	0	1	0
	phenylacetaldehyde dehydrogenase [EC:1.2.1.39]	R03300	11	13	16	1	3	7
	catechol 2,3-dioxygenase [EC:1.13.11.2]	R07795	23	64	60	35	8	15
	2-hydroxymuconate-semialdehyde hydrolase [EC:3.7.1.9]	R05865	6	5	10	4	0	5
	aliphatic nitrilase [EC:3.5.5.7]	R05358	18	31	9	17	4	11
	glutaconate CoA-transferase, subunit A [EC:2.8.3.12]	R05509	113	156	152	90	73	75
Monoterpenoid biosynthesis	(E)-8-carboxylinalool synthase [EC:1.14.14.-]	R09924	0	0	1	1	0	0
	geranyl diphosphate phosphohydrolase [EC:3.6.1.68]	R11551	0	1	0	0	0	0
Glyoxylate and dicarboxylate metabolism	acetyl-CoA synthetase [EC:6.2.1.1]; acetate/butyrate---CoA ligase [EC:6.2.1.1 6.2.1.2]	R01354	459	546	578	424	177	316
	acetyl-CoA synthetase [EC:6.2.1.1]; acetate/butyrate---CoA ligase [EC:6.2.1.1 6.2.1.2]	R01176	1	0	3	2	2	1
	malate synthase [EC:2.3.3.9]	R00472	51	105	143	90	22	49
	citrate synthase [EC:2.3.3.1]	R00351	173	235	372	209	55	147
	acetyl-CoA C-acetyltransferase [EC:2.3.1.9]	R01177	567	766	798	555	251	315
	methylmalonyl-CoA/ethylmalonyl-CoA epimerase [EC:5.1.99.1]	R09979	82	64	117	40	29	55
	catalase [EC:1.11.1.6]	R02670	320	251	195	194	82	133
	hydroxypyruvate reductase [EC:1.1.1.81]	R02527	11	24	26	15	6	17
	glutamate--glyoxylate aminotransferase [EC:2.6.1.4 2.6.1.2 2.6.1.44]	R00588	79	102	84	84	19	46
	glutamate synthase (ferredoxin) [EC:1.4.7.1]	R10086	228	281	271	281	112	306
	glycine cleavage system P protein (glycine dehydrogenase) [EC:1.4.4.2]	R03425	343	334	514	326	223	345
	2-hydroxy-3-oxopropionate reductase [EC:1.1.1.60]	R01747	98	120	95	77	48	79
	lactaldehyde dehydrogenase / glycolaldehyde dehydrogenase [EC:1.2.1.22 1.2.1.21]	R01446	4	8	4	18	2	2
	lactaldehyde reductase [EC:1.1.1.77]; glycolaldehyde reductase [EC:1.1.1.-]	R02257	10	7	3	2	7	8
	2-dehydro-3-deoxyphosphogluconate aldolase / (4S)-4-hydroxy-2-oxoglutarate aldolase [EC:4.1.2.14 4.1.3.42]	R05605	89	92	82	72	47	98
	4-hydroxy-2-oxoglutarate aldolase [EC:4.1.3.16]	R00471	30	12	14	8	16	12
	arylformamidase [EC:3.5.1.9]; kynurenine formamidase [EC:3.5.1.9]	R04911	62	63	90	32	13	24
Naphthalene degradation	naphthalene,NADH:oxygen oxidoreductase (1,2-hydroxylating) [EC:1.14.12.12]	R02968	11	7	14	13	8	7
	cis-1,2-dihydronaphthalene-1,2-diol:NAD+ 1,2-oxidoreductase [EC:1.3.1.29]	R04115	0	0	1	1	2	0
	2-hydroxy-2H-chromene-2-carboxylate---(3E)-4-(2-hydroxyphenyl)-2-oxobut-3-enoate isomerase [EC:5.99.1.4]	R05137	1	11	4	8	4	5
	(3E)-4-(2-hydroxyphenyl)-2-oxobut-3-enoate hydro-lyase [EC:4.1.2.45]	R05136	2	1	0	0	0	1
	salicylaldehyde:NAD+ oxidoreductase [EC:1.2.1.65]	R02941	0	1	0	0	0	0
Ethylbenzene degradation	naphthalene 1,2-dioxygenase subunit alpha/beta [EC:1.14.12.12]	R05424<br>R05425<br>R05426<br>R05427	11	7	14	13	8	7
	ethylbenzene:(acceptor) oxidoreductase [EC:1.17.99.2]	R05745	1	10	22	31	4	2
	acetophenone:carbon-dioxide ligase (ADP-forming) [EC:6.4.1.8]	R05453	1	1	1	1	0	0
	acetyl-CoA acyltransferase [EC:2.3.1.16]	R05506	124	200	288	164	58	84
	ethylbenzene dioxygenase subunit alpha/beta [EC:1.14.12.-]	R05440	7	5	10	11	5	11
	2,3-dihydroxyethylbenzene 1,2-dioxygenase [EC:1.13.11.-]	R05416	0	0	0	1	0	0
	2-hydroxy-6-oxo-octa-2,4-dienoate hydrolase [EC:3.7.1.-]	R05366	0	2	7	1	0	3

Supplemental Table 6 Gene hit number related to anaerobic degradation pathways and other various metabolic pathways associated with carbon conversion of soil organic carbon were obtained from metagenomes and compared to KEGG pathways for each core. Each enzyme gene was conditionally formatted to compare them across sites to display high (red), medium (white), and low (blue) gene abundance.

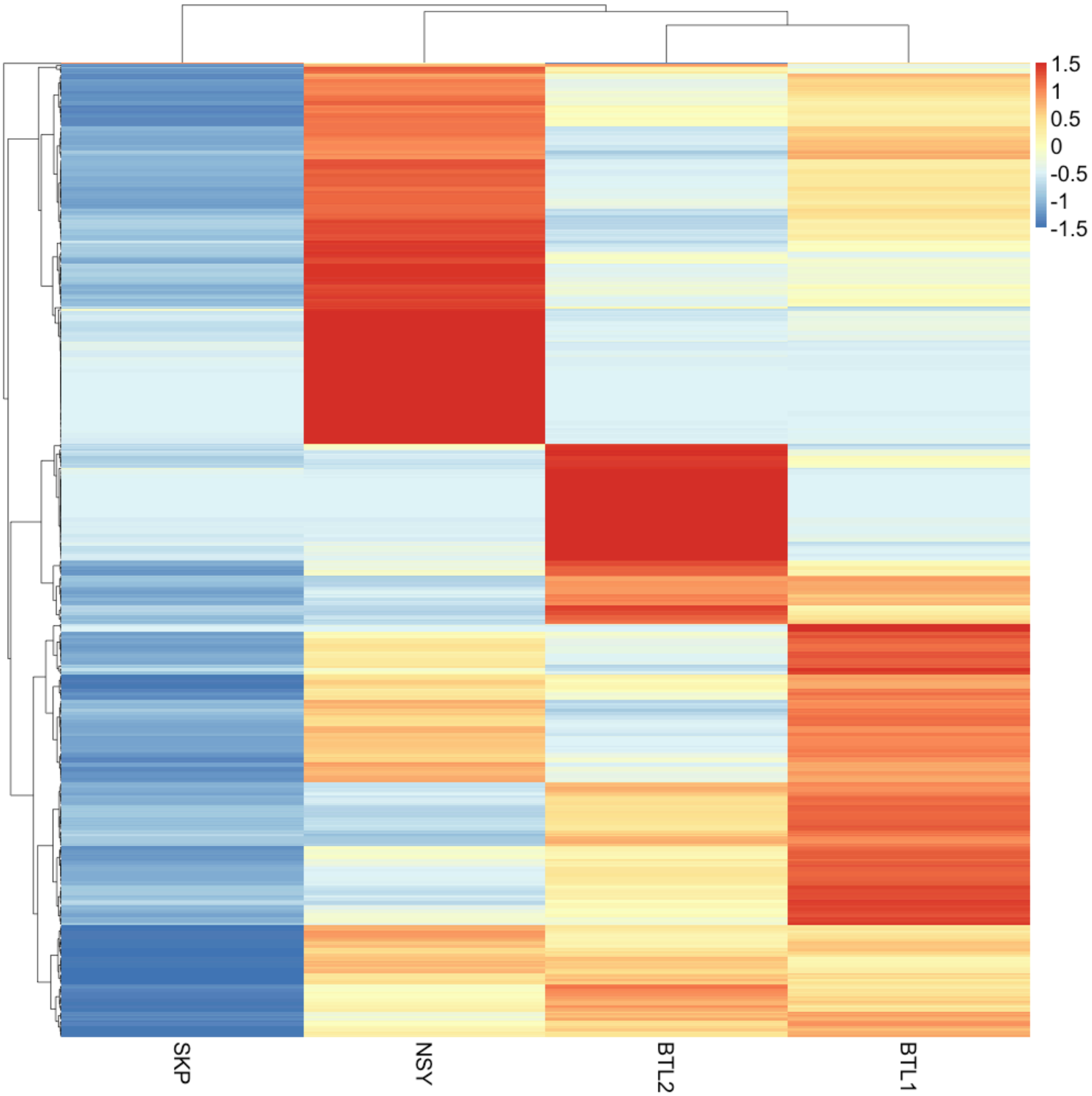

Supplemental Figure 5 Heatmap of total VOCs detected from each borehole. Differential VOC abundance was observed for each borehole.
